# Mice produce interneurons in the septum as a response to aversive experiences and antidepressant treatment

**DOI:** 10.1126/sciadv.aed3625

**Published:** 2026-06-10

**Authors:** Aikaterini Lampada, Chiara Rolando, Nigel Whittle, Anna Engler, Jens Tillmann, Joana Amorim Freire, Claudio Giachino, Elena Parmigiani, Ichiko Saotome, Angeliki Louvi, Jan Gründemann, Andreas Lüthi, Verdon Taylor

**Affiliations:** ^1^ Department of Biomedicine, University of Basel, Basel, 4058, Switzerland.; ^2^ Friedrich Miescher Institute for Biomedical Research, Basel, 4056, Switzerland.; ^3^ DZNE, Bonn, 53127, Germany.; ^4^ Departments of Neurosurgery and Neuroscience, Yale School of Medicine, New Haven, CT 06520, USA.; ^5^ Medical Faculty, University of Bonn, Bonn, Germany.; ^6^ University of Basel, Basel, Switzerland.

## Abstract

Adult neurogenesis sustains olfactory function, facilitates the acquisition of new memories, and provides resilience against depression in mice. Neural stem cells (NSCs) elicit selective responses to different physiological and pathological stimuli, thereby modulating neuron production. Here, we show that fear conditioning stimulates neurogenesis in the adult mouse brain septum by activating NSCs in the dorsal septum (dsNSCs) situated near a plexus of serotonergic axons originating from raphe nucleus neurons—key regulators of mood, anxiety, and stress responses. Elevation of serotonin (5HT) levels with the antidepressant and anxiolytic drug fluoxetine similarly promoted dsNSC proliferation and neurogenesis. The adult-born GABAergic interneurons integrate into septal nuclei. These findings suggest a potential adaptive response in mice to stress-inducing aversive experiences and elevated 5HT in the adult septum.

## Teaser

Aversive experiences and antidepressant treatment stimulate neuron production in the adult septum.

## INTRODUCTION

Neural stem cells (NSCs) in the mammalian brain undergo neurogenesis throughout adulthood to maintain homeostasis and physiological function in response to pathophysiological inputs (*1*, *2*). Neuron production occurs in adults of many vertebrate species, including fish, birds, rodents, primates, and probably in humans (*2*–*11*). Adult-born neurons produced in the ventricular-subventricular zone (V-SVZ) of the lateral ventricle walls and the subgranular zone of the hippocampal dentate gyrus (DG) integrate into established neuronal circuits in the olfactory bulb (OB) and DG, respectively. Intrinsic and extrinsic factors regulate adult neurogenesis, including by altering the equilibrium between signals that maintain NSC quiescence and those that induce activation (*1*, *12*, *13*).

Stress and antidepressants affect mood and behavior and modulate neurogenesis in the DG. Chronic stress suppresses neuron production in the DG by reducing proliferation (*14*–*18*), while antidepressants contribute to stress resilience and increase neuron production in the DG (*14*, *15*, *18*–*23*). Seratonin (5HT) neurotransmission is linked to stress, and agents that target the serotonergic system are commonly used to treat depression and anxiety (*24*). Selective serotonin reuptake inhibitors (SSRIs), which block the serotonin transporter (SERT) to increase extracellular 5HT levels, are the most widely used antidepressants (*25*). Although serotonergic neurons innervate many brain regions, the interplay between stress, SSRI treatment, and the regulation of neurogenesis has primarily been studied in the classic neurogenic niches, V-SVZ and DG (*14*–*23*). It remains unclear whether stress and antidepressant treatment affect neuronal production in other adult brain regions.

The brain septum is involved in several brain functions, including as part of the brain’s reward center, in stress, and in anxiety and is linked to learning and memory, social behavior, and fear but is also implicated in depression and schizophrenia (*26*–*32*). In rodents, the septum separates the lateral ventricles anteriorly and forms part of the roof of the third ventricle. The septum is divided into the lateral septum (LS) and medial septum. The LS lies directly adjacent to the lateral ventricles and is further subdivided into dorsal, intermediate, and ventral LS (fig. S1, A and B) (*30*, *31*). Here, we found that fear conditioning (FC) and stress, as well as SSRI treatment, trigger the production of new neurons in the septum of adult mice. We identified a population of NSCs in the dorsal lateral septum wall (LSW) and demonstrated their activation and production of new inhibitory interneurons, which migrate to septal nuclei.

## RESULTS

### FC induces proliferation and neurogenesis in the dorsal LSW

**Fig. 1.**
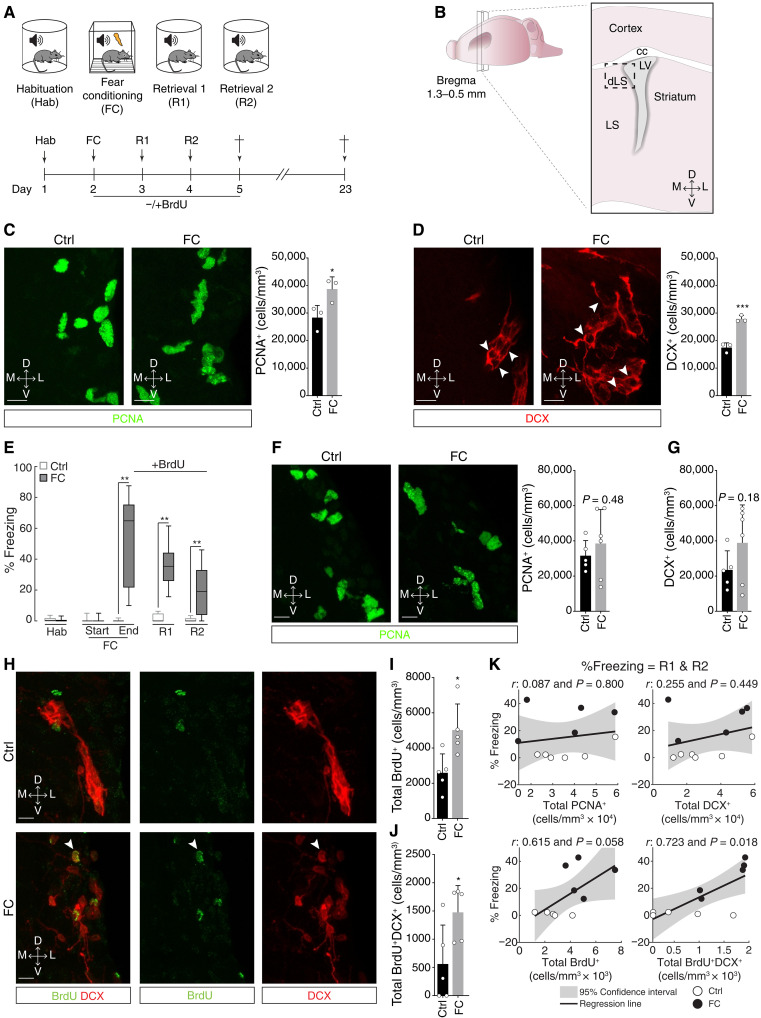
FC induces neurogenesis in the dorsal LS. (**A** and **B**) Scheme of the FC paradigms and mouse brain (septum shaded) with section showing the dorsal lateral septum (dLS; dotted rectangle). (**C** and **D**) Images and quantifications of PCNA^+^ cells and DCX^+^ neuroblasts (arrowheads) in the dorsal LSW (day 5). Control (Ctrl) animals did not receive foot shocks. Bars represent means ± SD of *N* = 3. Statistical significance was calculated by an unpaired *t* test: *P < 0.05 and ***P < 0.001 (PCNA^+^: *t* = 2.888, df = 4, and Cohen’s *d* = 2.36; DCX^+^: *t* = 8.703, df = 4, and Cohen’s *d* = 7.10). (**E**) Quantification of freezing response of mice across habituation (Hab), FC (start and end), R1, and R2 phases. *N* = 9 FC and *N* = 10 Ctrl. Median (line), interquartile range (box), and full data range (whiskers). Statistical significance was calculated by Wilcoxon signed-rank test: **P < 0.01. (**F**) Images and quantifications of PCNA^+^ cells in the dorsal LSW (day 23). Bars represent means ± SD of *N* = 5 Ctrl and *N* = 6 FC. Statistical significance was calculated by an unpaired *t* test: (PCNA^+^: *t* = 0.7423 and df = 9). (**G**) Quantification of DCX^+^ neuroblasts in the dorsal LSW (day 23). Bars represent means ± SD of *N* = 5 Ctrl and *N* = 6 FC. Statistical significance was calculated by an unpaired *t* test: (DCX^+^: *t* = 1.444 and df = 9). (**H**) Images of BrdU^+^ and DCX^+^ cells in the dorsal LSW (day 23). (**I** and **J**) Quantification of BrdU^+^ and BrdU^+^DCX^+^ cells in the dorsal LSW (day 23). Bars represent means ± SD of *N* = 5. Statistical significance was calculated by an unpaired *t* test: *P < 0.05 (BrdU^+^: *t* = 2.962 and df = 8; BrdU^+^DCX^+^: *t* = 2.437 and df = 8). (**K**) Scatterplots showing freezing behavior during R1 and R2 and the expression of PCNA, DCX, and BrdU (day 23). For PCNA and DCX, *N* = 6 Ctrl and *N* = 5 FC; for BrdU and BrdU/DCX, *N* = 5 Ctrl and FC. Spearman’s correlation coefficient (*r*)*.* Scale bars, 10 μm. cc, corpus callosum; D, dorsal; V, ventral; M, medial; L, lateral orientation of the mouse brain.

Adult-born neurons contribute to stress resilience (*20*, *33*–*38*) and the modulation of anxiety-like behaviors (*18*, *21*, *33*, *39*–*43*). We performed an auditory FC paradigm in which mice learn to associate an initially neutral auditory conditioned stimulus (CS) with an aversive unconditioned foot shock (US) (Fig. 1A) (*44*–*46*). After FC, mice exhibited elevated freezing behavior in response to CS exposure compared to control (Ctrl) mice that were exposed to CS only but not to the US (fig. S1C).

One day after FC, proliferating cell nuclear antigen–positive (PCNA^+^) mitotic cells were increased in the dorsal LSW (FC: 38738.51 ± 4392.32 PCNA^+^ cells/mm^3^, Ctrl: 28355.63 ± 4414.21 PCNA^+^ cells/mm^3^) (Fig. 1, A to C, and fig. S1D). In addition, doublecortin-positive (DCX^+^) neuroblasts were increased in the subependymal layer of the dorsal LSW of FC mice (FC: 27912.75 ± 1208.94 DCX^+^ cells/mm^3^, Ctrl: 17499.53 ± 1683.33 DCX^+^ cells/mm^3^) (Fig. 1D and fig. S1E). Therefore, FC promoted proliferation and the production of immature neurons (i.e., neurogenesis) in the dorsal LSW.

We analyzed the dynamics of the acute FC-induced response and assessed the persistence of elevated proliferation and neurogenesis in the dorsal LSW. Eighteen days after the FC, proliferation in dorsal LSW of FC mice had returned to Ctrl levels (FC: 38788.23 ± 19236.50 PCNA^+^ cells/mm^3^, Ctrl: 31845.24 ± 8616.17 PCNA^+^ cells/mm^3^) (Fig. 1, E and F, and fig. S2, A and B). DCX^+^ neuroblasts were also no longer increased in the dorsal LSW of FC mice compared to Ctrl mice (FC: 38904.24 ± 21547.69 DCX^+^ cells/mm^3^, Ctrl: 23495.43 ± 10894.84 DCX^+^ cells/mm^3^; *P* = 0.18) (Fig. 1G and fig. S2C).

To trace the fate of the mitotic cells in the dorsal LSW, we used thymidine analog labeling and treated mice with 5-bromo-2′-deoxyuridine (BrdU) for 3 days during the FC paradigm (Fig. 1A and fig. S2D). BrdU^+^ cells were increased in the dorsal LSW of FC mice after 18 days (FC: 5020.55 ± 1481.42 BrdU^+^ cells/mm^3^, Ctrl: 2599.05 ± 1071.50 BrdU^+^ cells/mm^3^), confirming that FC-induced proliferation and some cells remained in the dorsal LSW (Fig. 1, H and I, and fig. S2E). BrdU^+^DCX^+^ newborn neuroblasts were increased in the dorsal LSW of FC mice compared to Ctrl mice (FC: 1475.68 ± 476.09 cells/mm^3^, Ctrl: 562.12 ± 689.89 cells/mm^3^), indicating an acute production of neuroblasts from mitotic FC-stimulated cells (Fig. 1, H and J, and fig. S2F).

We investigated the relationship between the freezing behavior of mice during memory retrieval phases and the increase in proliferation and neurogenesis markers in the dorsal LSW (Fig. 1K and fig. S2G). The FC-induced increase in mitotic cells (total BrdU^+^ cells/mm^3^) in the dorsal LSW correlated with freezing behavior during retrieval 1 (R1) and retrieval 2 (R2). In addition, the FC-induced increase in newborn neuroblasts (total BrdU^+^DCX^+^ cells/mm^3^) positively correlated with freezing during R1 and R2 (Fig. 1K). This suggests that stress-inducing aversive experiences increase neurogenesis in the dorsal LSW.

### The adult dorsal LSW contains neurogenic stem cells

**Fig. 2.**
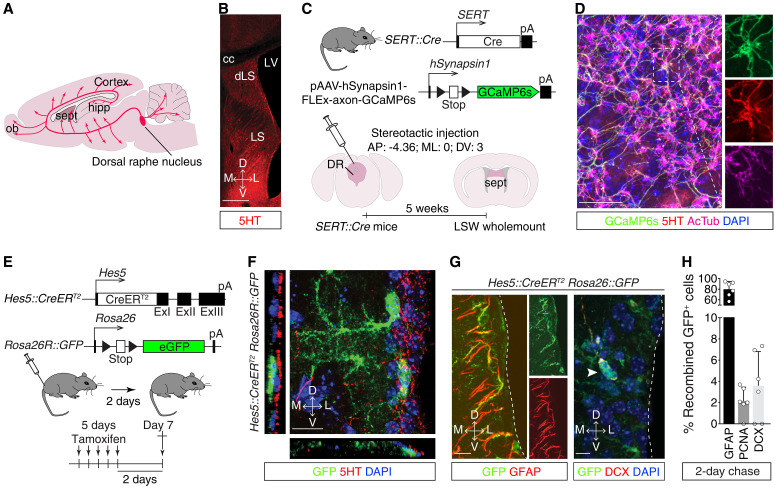
Dorsal LSW contains quiescent NSCs in close proximity to 5HT axons. (**A**) Scheme of dorsal raphe (DR) nucleus serotonergic neuron innervation of the adult mouse brain (red arrows). (**B**) Image showing 5HT^+^ afferents in the LS. (**C**) Scheme of *SERT::Cre* allele, pAAV-hSynapsin1-FLEx-axon-GCaMP6s viral construct, and coordinates of DR injection with timeline. (**D**) Image of a ventricular septum wall wholemount with 5HT^+^ DR-derived axons (GCaMP6s^+^) and acetylated tubulin (AcTub) staining of ependymal cilia. (**E**) *Hes5::CreER^T2^* transgene and *Rosa26R::GFP* Cre-reporter allele with exons (Ex) and poly-adenylation (pA) sites. Lineage tracing timeline; tamoxifen was administered intraperitoneally. (**F**) Orthogonal reconstruction (*Y*-*Z* and *X*-*Z* axis) of confocal images showing 5HT^+^ axons and NSCs (GFP^+^) in the dorsal LSW. (**G**) Images showing *Hes5::CreER^T2^*-derived GFP^+^GFAP^+^ and GFP^+^DCX^+^ cells and (**H**) quantifications of GFAP^+^,PCNA^+^ and DCX^+^ cells in the dorsal LSW (day 7). Bars represent mean ± SD of *N* = 6. Scale bars, 100 μm (B), 50 μm (D), 10 μm (F), and 15 μm (G). ob, olfactory bulb; hipp, hippocampus; cc, corpus callosum; sept, septum; dLS, dorsal lateral septum; DR, dorsal raphe; LSW, lateral septal wall; LV, lateral ventricle; 5HT, D, dorsal; V, ventral; M, medial; L, lateral orientation of the mouse brain.

The serotonergic system of the dorsal raphe plays a key role in stress and anxiety, and FC increases serotonergic neuron activity in the dorsal raphe (*47*–*49*). Serotonergic neurons innervate many brain regions, including the septum (Fig. 2, A and B) (*50*, *51*). 5HT^+^ axons are distributed throughout the dorsal, intermediate, and ventral LS and also localize to the LSW (Fig. 2B and fig. S1B). Wholemount preparations of the septum revealed 5HT^+^ axons on the LSW ependyma surface inside the lateral ventricle (fig. S3, A and B). We addressed whether these axons originate from serotonergic neurons located in the raphe nuclei (*52*, *53*). We labeled dorsal raphe serotonergic neurons by infection of *SERT::Cre* mice with a virus encoding Synapsin1 FLEx-axon-GCaMP6s (Fig. 2C and fig. S3C) (*54*). GCaMP6s^+^ 5HT^+^ axons covered the surface of the LSW in close apposition to acetylated tubulin-labeled cilia of the ependyma cells showing that serotonergic neurons in the dorsal raphe are a source of these axons (Fig. 2D and fig. S3D). Analysis of proteomic data from the LSW revealed components of the 5HT biosynthesis pathway, thereby independently confirming the presence of serotonergic axons and 5HT production in the LSW (fig. S3, E and F) (*55*).

We hypothesized that the dorsal LSW contains stem/progenitor cells that are activated by stressful experiences and give rise to new neurons. NSC and neurogenesis-associated proteins are present in the LSW (fig. S4A) (*55*). The Notch signal target *Hes5* is expressed by NSCs in the adult brain (*56*–*59*). To address whether the dorsal LSW contains NSCs, we genetically labeled Hes5*^+^* cells in the LSW of *Hes5::CreER^T2^ Rosa26R::GFP* mice (Fig. 2E) (*58*). *Hes5::CreER*^*T2*+^ cells [green fluorescent protein–positive (GFP^+^)] showed NSC-like morphologies and were embedded in a V-SVZ–like structure in close proximity to the plexus of 5HT^+^ axons covering the LSW surface (Fig. 2F). Most *Hes5::CreER*^*T2*+^ cells in the dorsal LSW had a radial NSC morphology and expressed glial fibrillary acidic protein (GFAP) (79.98 ± 14.80%) (Fig. 2, G and H). The LSW contains PCNA^+^ mitotic cells (28355.63 ± 4414.21 cells/mm^3^) (Fig. 1C and fig. S1D), but most *Hes5::CreER^T2+^* cells (GFP^+^) were mitotically inactive (PCNA^+^GFP^+^: 2.16 ± 1.31% of GFP^+^ cells in LSW) (Fig. 2H) consistent with them being quiescent NSCs. Very few *Hes5::CreER^T2^*-derived cells (GFP^+^) expressed DCX^+^ 2 days post-labeling (DCX^+^GFP^+^: 3.59 ± 3.22% of GFP^+^) (Fig. 2, G and H).

**Fig. 3.**
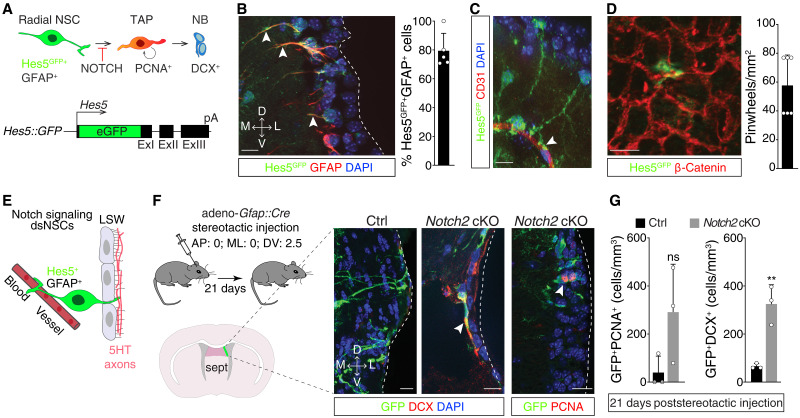
Dorsal LSW NSCs depend on Notch signaling. (**A**) Scheme of adult neurogenesis showing radial NSCs, transient amplifying progenitors (TAPs), and neuroblasts (NBs). *Hes5::GFP* transgene with exons (Ex) and poly-adenylation (pA) sites. (**B**) Image of radial Hes5^GFP+^GFAP^+^ cells (arrowheads) and quantification (percent Hes5^GFP+^GFAP^+^/Hes5^+^) in the dorsal LSW. Bar represents mean ± SD of *N* = 5. (**C**) Image of radial Hes5^GFP+^ cells and blood vessel (CD31^+^ arrowhead) in the dorsal LSW. (**D**) Image of Hes5^GFP+^ cells in wholemount protruding between the LSW ependyma (adherens junctions labeled by β-catenin). Quantification of Hes5^GFP+^ cell–containing pinwheels in the dorsal LSW. Bar represents mean ± SD of *N* = 6. (**E**) Scheme of the dorsal LSW niche with radial Hes5^GFP+^GFAP^+^ dsNSC, blood vessel, ependymal cells (gray) and 5HT axons. (**F**) Experimental procedure and brain coordinates of stereotactic adeno-*Gfap::Cre* injections into the dorsal LS of *Rosa26R*- *GFP* Cre-reporter mice. Images of GFP^+^DCX^+^ neuroblasts and GFP^+^PCNA^+^ cells generated by adeno-*Gfap::Cre*-infected NSC in the dorsal LSW (21 days poststereotactic injection). (**G**) Quantification of GFP^+^PCNA^+^ and GFP^+^DCX^+^ cells in the dorsal LSW (21 days poststereotactic injection). Bars represent mean ± SD of *N* = 3 for both Ctrl and *Notch2* cKO. Statistical significance was calculated by an unpaired *t* test: ns, not significant; **P < 0.01 (GFP^+^DCX^+^: *t* = 5.519, df = 4, and Cohen’s *d* = 4.49; GFP^+^PCNA^+^: *t* = 2.061, df = 4, and Cohen’s *d* = 1.69). Scale bars, 15 μm [(B) to (D)] and 25 μm (F). sept, septum; LSW, lateral septal wall; D, dorsal; V, ventral; M, medial and L, lateral orientation of the mouse brain.

In the V-SVZ of the lateral ventricles, quiescent radial NSCs (B cells) sporadically enter cell division to generate transient amplifying progenitor cells (C cells), which produce DCX^+^ neuroblasts (A cells) (Fig. 3A) (*2*). Notch signaling maintains V-SVZ NSCs and regulates their differentiation (*56*, *60*–*64*). Therefore, we analyzed Notch signaling in the LS. Recombination signal binding protein for immunoglobulin kappa J region (RBPj) is a pivotal nuclear component of the Notch pathway, and transcription factors of the HES and HEY families are effectors of Notch signaling (fig. S4B) (*65*, *66*). *Hes5::GFP* (Hes5^GFP^) mice report canonical Notch signaling and label NSCs in the adult neurogenic niches (Fig. 3B) (*56*–*58*). RBPj^+^ cells in the LSW with radial NSC-like morphology expressed Hes5^GFP^ (fig. S4C). The radial NSCs expressed GFAP, and most exhibited Notch activity (GFAP^+^Hes5^GFP+^ 79.45 ± 12.05% of Hes5^GFP+^) (Fig. 3B). Hes5^GFP+^ radial cells contacted blood vessels in the septal V-SVZ parenchyma (Fig. 3C) and the ventricle through pinwheel-like structures in the ependymal lining (57.69 ± 21.07 pinwheels/mm^2^) (Fig. 3D and fig. S4, D and E). Therefore, radial GFAP^+^ Hes5^GFP+^ cells in the LSW [hereafter referred to as dorsal septal NSCs (dsNSCs)] share characteristics with the B cells (NSCs) in the V-SVZ of the forebrain lateral ventricles and are in close proximity to a plexus of 5HT^+^ axons covering the ependyma surfaces of the LSW (Fig. 3E).

Infusion of the antimitotic agent cytosine arabinoside (AraC) into the lateral ventricles kills mitotic progenitors and blocks neurogenesis in the V-SVZ of the lateral wall of the lateral ventricles (*67*). AraC treatment did not affect the number of the putative dsNSCs in the LSW, confirming their mitotic inactivity (fig. S4, F to H). The production of new neurons in the adult brain is reduced during aging, and this correlates with exhaustion of the proliferating NSC pool (*56*, *57*, *68*). Hes5^GFP+^ dsNSCs in the dorsal LSW remained constant between 8 and 52 weeks of age, confirming the relative quiescence of this population in sedentary mice (fig. S4I).

To determine whether dsNSCs are dependent on Notch activity, we conditionally knocked out (cKO) either *Rbpj* or both *Notch1* and *Notch2* from *Hes5::CreER*^*T2*+^ cells in the dorsal LSW and followed their fate (fig. S5). Both *Rbpj* cKO and *Notch1*, *Notch2* double cKO resulted in an increase in proliferation (PCNA^+^ cells) and enhanced neuroblast (DCX^+^) production in the dorsal LSW (fig. S5, C, D, G, and H). Concomitant deletion of *Notch1* and *Notch2* recapitulated the *Rbpj* cKO phenotype, demonstrating that dsNSCs rely on active canonical Notch signaling to maintain quiescence and inhibit entry into neurogenesis.

NOTCH2 controls cell cycle entry of adult NSCs in the lateral walls of the lateral ventricles and the DG (*61*, *69*). Genetic labeling in *Notch2::CreER*^*T2*-*SAT*^ *Rosa26R::tdTomato* mice showed that radial GFAP^+^ dsNSCs express *Notch2* (fig. S6, A and B). *Notch2* cKO (*Notch2^lox/lox^ Rosa26R::GFP*) increased proliferation (GFP^+^PCNA^+^) and neuroblast (GFP^+^DCX^+^) production in the dorsal LSW (fig. S6, C to F). Thus, radial Hes5^+^ cells in the LSW have quiescent NSC characteristics, and their proliferation is regulated by NOTCH2. To assess whether the neuroblasts in the dorsal LSW were derived from local NSCs, we restricted the *Notch2* cKO to dorsal LSW GFAP^+^ cells by stereotactic injection of adeno-*Gfap::Cre* virus into the dorsal septum of adult *Notch2^lox/lox^ Rosa26R::GFP* mice (Fig. 3F and fig. S7A) (*56*, *70*). Adeno-*Gfap::Cre*–induced genetic recombination was restricted to GFAP^+^ cells of the dorsal LSW (fig. S7B). Upon *Notch2* cKO, adeno-*Gfap::Cre* infected GFAP^+^ cells in the dorsal LSW entered cell cycle and generated neuroblasts (Fig. 3, F and G, and fig. S7C).

Neuroblasts generated in the V-SVZ of the lateral ventricle wall migrate to the OB (*71*–*73*). Lineage tracing of dsNSCs in the dorsal LSW infected with adeno-*Gfap::Cre* showed that the locally generated dorsal septal neuroblasts do not migrate to the OB (fig. S7, D and E). Thus, the dorsal LSWs harbor NSCs with latent neurogenic potential that do not give rise to OB neurons.

### Fluoxetine increases proliferation and neurogenesis in the dorsal LSW

dsNSCs are close to a plexus of raphe nuclei–derived 5HT^+^ axons covering the ependyma surfaces of the LSW. As FC induced neurogenesis in the LSW and stress activates 5HT neurons in the raphe nuclei, we hypothesized that dsNSCs are activated by stress-induced 5HT signaling to give rise to new neurons in the LS. 5HT binds two families of receptors (fig. S8A). To investigate whether dsNSCs are receptive to 5HT, we sorted *Hes5^GFP+^* dsNSCs from the dorsal LSW and examined their expression of all 14 5HT receptors (fig. S8, A to C). dsNSCs expressed the metabotropic receptors *5Htr1a*, *5Htr1f*, *5Htr2a*, *5Htr2b*, *5Htr4*, *5Htr5a*, *5Htr6*, and *5Htr7*, but not *5Htr3* ionotropic receptors (fig. S8, A and D). Therefore, dsNSCs could be directly regulated through 5HT receptor–dependent signaling.

Fluoxetine blocks the SERT, leading to increased levels of 5HT in the brain (*25*). Functional magnetic resonance imaging analysis of 5HT transport in the rat brain showed reuptake inhibition by fluoxetine to be highest in the rostral dorsal LS (*74*). We hypothesized that quiescent dsNSCs could respond to increased 5HT levels during fluoxetine treatment. Activated NSCs in the V-SVZ express brain lipid-binding protein (BLBP), which, unlike Hes5 expression, is retained by proliferative progenitors (C cells) (*56*). We treated *Hes5::GFP*, *BLBP::mCherry* mice with fluoxetine for 7 days and analyzed the effects in the LS, 2 and 19 days post-treatment (fig. S8E). Fluoxetine acutely increased the generation of mitotic cells (PCNA^+^) and neuroblasts (DCX^+^) in the dorsal LSW (fig. S8F). In addition, fluoxetine increased the number of BLBP^mCherry+^ cells and decreased the total number of Hes5^GFP+^ cells in the dorsal LSW (fig. S8G). This implies a reduction in dsNSCs at the expense of the generation of proliferating transient intermediate progenitors.

**Fig. 4.**
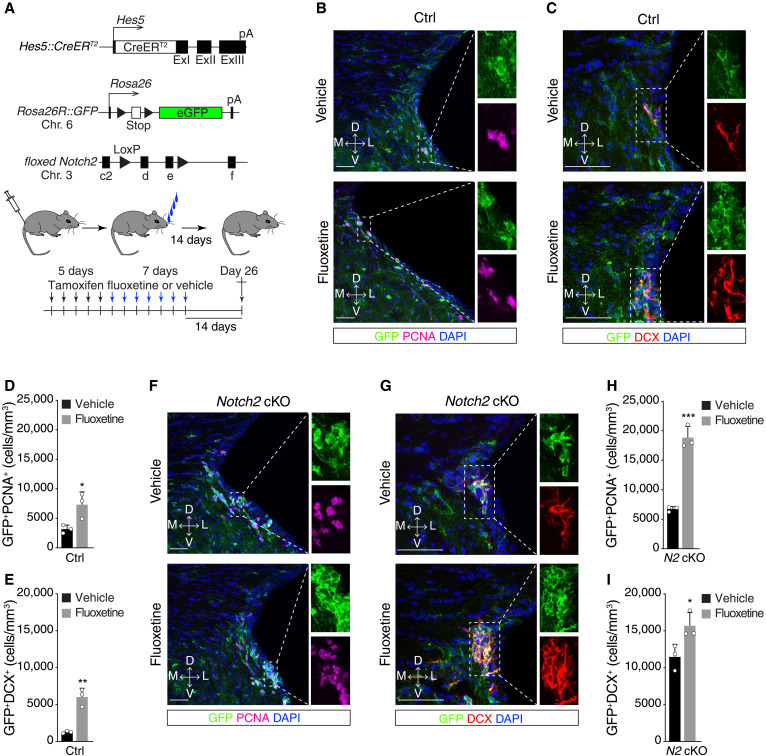
Fluoxetine induces neurogenesis in the dorsal LSW. (**A**) Floxed *Notch2*, *Hes5::CreER^T2^* transgene and *Rosa26R::GFP* Cre-reporter allele with chromosome (Chr.), exons (Ex), LoxP, and poly-adenylation (pA) sites. Lineage tracing timeline; tamoxifen was administered intraperitoneally and vehicle/fluoxetine per os (p.o.). (**B** and **C**) Images of serial confocal sections showing *Hes5::CreER^T2^*-derived PCNA^+^ cells (B) and DCX^+^ neuroblasts (C) in the dorsal LSW (day 26). Dotted rectangles mark the magnified regions of interest. (**D** and **E**) Quantification of *Hes5::CreER^T2^*-derived (GFP^+^) mitotic cells (PCNA^+^; D) and neuroblasts (DCX^+^; E) in the dorsal LSW of Ctrl animals treated with fluoxetine or vehicle. Bars represent mean ± SD of *N* = 3 mice per group. Statistical significance was calculated by an unpaired *t* test: *P < 0.05 and **P < 0.01 [for (D): Ctrl, *t* = 3.182 and df = 4; for (E): Ctrl, *t* = 6.821 and df = 4). (**F** and **G**) Images of serial confocal sections showing *Hes5::CreER^T2^*-derived PCNA^+^ cells (F) and DCX^+^ neuroblasts (G) in the dorsal LSW (day 26). Dotted rectangles mark the magnified regions of interest. (**H** and **I**) Quantification of *Hes5::CreER^T2^*-derived (GFP^+^) mitotic cells (PCNA^+^; H) and neuroblasts (DCX^+^; I) in the dorsal LSW (day 26). Bars represent mean ± SD of *N* = 3 mice per group. Statistical significance was calculated by an unpaired *t* test: *P < 0.05 and ***P < 0.001 [for (H): *Notch2* cKO, *t* = 11.47 and df = 4; for (I): *Notch2* cKO, *t* = 2.940 and df = 4]. Scale bars, 50 μm [(B), (C), (F), and (G)]. D, dorsal; V, ventral; M, medial and L, lateral orientation of the mouse brain.

We traced the fate of fluoxetine-activated Hes5^+^ dsNSCs in *Hes5::CreER*^*T2*+^ *Rosa26R::GFP* mice. We genetically labeled *Hes5::*^+^ dsNSCs (GFP^+^) and subsequently treated the mice with fluoxetine for 7 days (Fig. 4A). Fluoxetine increased Hes5^+^ dsNSC generation of mitotic progenitors (GFP^+^PCNA^+^), neuroblasts (GFP^+^DCX^+^), and neurons (GFP^+^NeuN^+^) (Fig. 4, B to E, and fig. S9, A to D). Therefore, reducing 5HT uptake increased neurogenesis in the dorsal LSW from Hes5^+^ dsNSCs.

We addressed a putative crosstalk between Notch signaling and fluoxetine in dsNSC activation by *Notch2* cKO (*Hes5::CreER*^*T2*+^ *Notch2 ^lox/lox^ Rosa26R::GFP*) combined with fluoxetine treatment. Combined *Notch2* cKO and fluoxetine treatment increased proliferation and neuroblast formation in the dorsal LSW over and above *Notch2* cKO or fluoxetine treatment alone (Fig. 4, F to I, and fig. S9, E to G). The combined effects of *Notch2* cKO and fluoxetine on proliferation were additive (Fig. 4, B to I, and fig. S9 A, B, E, and F). However, this additive effect on proliferation did not result in an increase in newborn neurons (GFP^+^NeuN^+^) in the dorsal LSW (fig. S9G). This indicates that, similar to its effects on DG neurogenesis, fluoxetine affects neurogenesis in the LSW by increasing the proliferation of activated progenitors and does not induce neuronal differentiation (*22*). We examined whether fluoxetine treatment directly affects the level of Notch signal activity in dsNSCs. Fluorescence-activated cell sorting (FACS) analysis of dsNSCs showed no change in Hes5^GFP^ expression levels upon fluoxetine treatment in vivo (fig. S9H). Thus, fluoxetine likely activates dsNSCs through a Notch-independent mechanism. Overall, our data indicate that the fluoxetine-mediated increase in 5HT levels, potentially released from axons in the proximity of the LSW, increases neurogenesis by acting on dsNSCs.

### The LSW generates new LS neurons

**Fig. 5.**
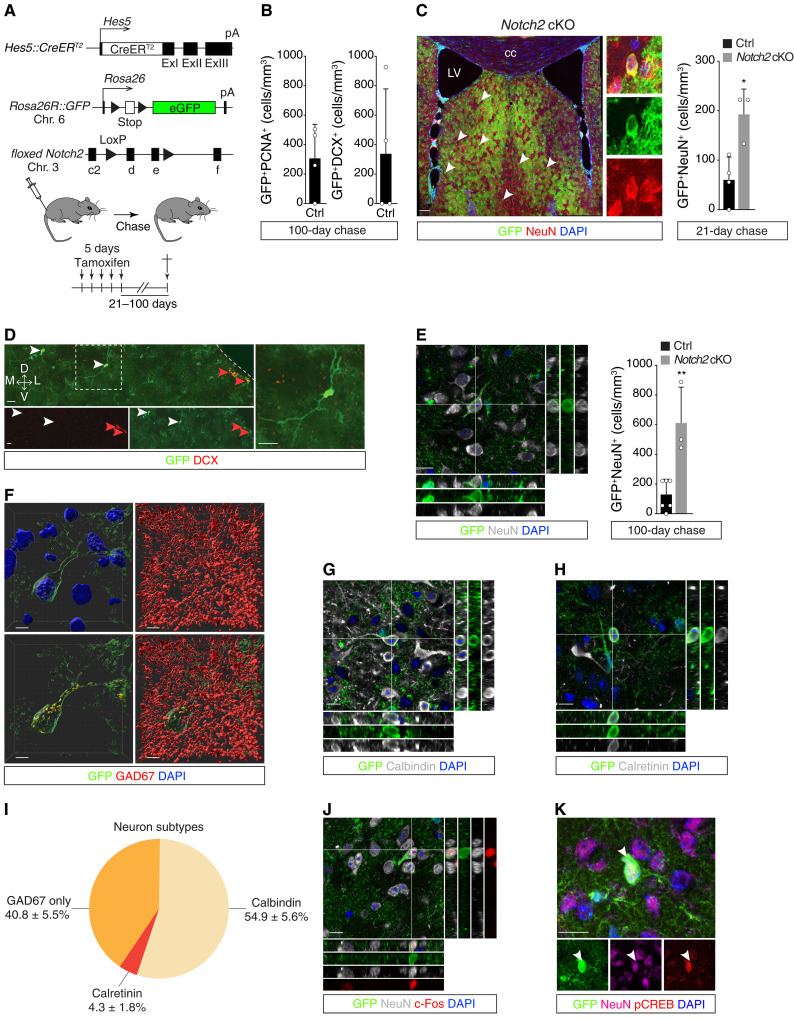
Dorsal LSW NSCs generate local GABAergic interneurons. (**A**) Floxed *Notch2* allele, *Hes5::CreER^T2^* transgene, and *Rosa26R::GFP* Cre-reporter allele. Lineage tracing timelines following tamoxifen administration. (**B**) Quantification of GFP^+^PCNA^+^ cells and GFP^+^DCX^+^ neuroblasts in the dorsal LSW (100 days Ctrl). Bars represent mean ± SD of *N* = 4. (**C**) Image of GFP^+^NeuN^+^ neurons in the LS (21 days *Notch2* cKO). Quantification of GFP^+^NeuN^+^ neurons in the LS (21 days). Bars represent mean ± SD of *N* = 4 Ctrl and *N* = 3 *Notch2* cKO. Statistical significance was calculated by an unpaired *t* test: *P < 0.05 (*t* = 3.584 and df = 5). (**D**) Image of *Notch2* cKO-derived neurons (white arrowheads) and neuroblasts (DCX^+^) in the dorsal LSW (red arrowheads). (**E**) Orthogonal reconstruction (separate channels in *Y*-*Z* and *X*-*Z* axes) of confocal images depicting a *Hes5::CreER^T2^*-derived neuron (GFP^+^NeuN^+^) in the LS (100 days *Notch2* cKO) and quantification (100 days). Bars represent mean ± SD of *N* = 6 Ctrl and *N* = 3 *Notch2* cKO. Statistical significance was calculated by an unpaired *t* test: **P < 0.01 (*t* = 4.359 and df = 7). (**F**) 3D reconstruction (Imaris) of a GAD67^+^ *Hes5::CreER^T2^*-derived (GFP^+^) neuron in the LS (100 days *Notch2* cKO). (**G** and **H**) Orthogonal reconstruction (separate channels in *Y*-*Z* and *X*-*Z* axes) of confocal images of *Hes5::CreER^T2^*-derived (GFP^+^) calbindin^+^ (G) and calretinin^+^ (H) neurons in the LS (100 days *Notch2* cKO). (**I**) Quantification of *Hes5::CreER^T2^*-derived neuronal subtypes in the LS (100 days). (**J**) Orthogonal reconstruction (separate channels in *Y*-*Z* and *X*-*Z* axes) of confocal images showing a *Hes5::CreER^T2^*-derived (GFP^+^) c-Fos^+^ NeuN^+^ neuron in the LS (100 days *Notch2* cKO). (**K**) Image of *Hes5::CreER^T2^*-derived phosphoCREB^+^ (pCREB) NeuN^+^ neuron (arrowheads) in the septum (21 days). Scale bars, 100 μm [(C), (D), (E), (G), (H), and (J)], 5 μm (F), and 10 μm (K).

We addressed the long-term neurogenic potential of dsNSCs by genetic lineage tracing (Fig. 5A). *Hes5::CreER*^*T2*+^ dsNSCs continued to generate proliferative progenitors and neuroblasts in the LS for more than 100 days, consistent with being long-term NSCs (Fig. 5B and fig. S9I). *Notch2* cKO notably increased the number of newly generated neurons (GFP^+^NeuN^+^), which accumulated in septal nuclei over time (Fig. 5, C to E, and fig. S9, J and K). All adult-born septal neurons (GFP^+^NeuN^+^) expressed GAD67 (100 ± 0%), and many coexpressed calbindin (54.9 ± 5.6%). A few newborn neurons expressed calretinin (4.3 ± 1.8%) (Fig. 5, F to I). This is consistent with most neurons in the LS being GABAergic (*75*). Newborn GFP^+^NeuN^+^ neurons integrated into septal nuclei, and some expressed nuclear phosphoCREB and c-Fos, consistent with being synaptically and electrically active (Fig. 5, J and K) (*76*–*78*). Thus, activated dsNSCs generate local GABAergic interneurons.

## DISCUSSION

The LS processes emotional information and modulates behavioral responses to stress and stress-induced social dysfunction. Septal functions in stress are resolved at the level of neural circuits between the septum and the hippocampus, hypothalamus, and amygdala (*26*–*29*, *79*–*82*). Here, we describe a latent neurogenic niche in the dorsal LS that generates new septal GABAergic interneurons in adult mice. The LSW niche includes 5HT axons, and resident dsNSCs respond to FC and antidepressant fluoxetine treatment by entering the cell cycle and increasing the generation of adult-born neurons. Although the generation of OB neurons has been described in the ventral septal wall (*71*, *72*, *83*), and gliogenic stem cells were identified in the region (*84*), neurogenic NSCs in the dorsal LSW and the production of newborn septal neurons have not been described previously. One likely explanation is that the dsNSCs are dormant in sedentary mice, and the number of newly generated neurons is minimal under normal conditions. We initially identified this neurogenic zone due to the activation of the NSCs following FC and confirmed their presence by Notch signal ablation and the presence of Hes5^+^ radial cells in a stem cell–like niche. Detailed analysis of the niche revealed the plexus of serotonergic axons emanating from the dorsal raphe nucleus which is a pivotal station in the stress network of the brain. Our findings that FC and SSRI antidepressant treatment stimulate the latent neurogenic program in the dorsal LS of adult mice (fig. S10) suggest that these newborn neurons could potentially be associated with septal functions in response to stress-inducing aversive experiences. As the classic FC paradigm used here does not allow separation of stress-related effects from learning-associated plasticity, we cannot exclude that the neurogenic response stimulated by FC reflects a combination of fear-associated learning and stress.

We did not observe newly generated NeuN^+^ neurons in the septum of FC mice. In the adult DG and central olfactory system, the maturation of newborn neurons generated by NSCs can take weeks (*56*, *58*). Hence, mature NeuN^+^ neurons may only appear weeks after FC. Alternatively, the increase in neuroblast generation in the dorsal LSW in response to FC may not translate into neuronal differentiation and maturation. The survival, fate, maturation, and circuit integration of the newly generated neuroblasts following FC remain to be determined. However, our data suggest that the transient stress of the FC paradigm and the transitory increase in progenitor proliferation and neuroblast production in the dorsal LSW are not an immediate adaptation to the conditioning. We postulate that the transient proliferative response reflects activation of dormant dsNSCs rather than a sustained neurogenic program directly linked to learning. It will be interesting to determine, using sequential DNA analog labeling, whether the stem cell divisions are self-renewing and maintain the NSC pool, or whether the stem cells are depleted following FC stimulation. Although the newborn septal neurons may contribute to an adaptation to stressful experiences, it cannot be excluded that they are a maladaptive response.

Stress blocks neurogenesis in the DG, whereas fluoxetine induces proliferation and increases neuron production (*14*, *15*, *19*, *20*, *22*). Therefore, the increased LSW neurogenesis we observed following FC seems contradictory. However, chronic and acute stress differentially affects neuron production in the adult brain. Decreased neurogenesis in the DG was mainly observed during chronic stress and has been linked to elevated glucocorticoid signaling (*85*–*87*). Acute stress, on the other hand, either increases neurogenesis in the adult hippocampus or does not have an effect (*88*). Similarly, chronic but not acute foot shock stress suppresses proliferation in the rat DG (*89*). Thus, the increase in LSW neurogenesis observed following FC might reflect a region-specific response to an acute stress. The newborn neurons in the septum are GABAergic compared to the glutamatergic neuron fate of adult DG born neurons, which implies distinct patterning and specification of the stem cells in these regions. This could also explain the distinctive neurogenic characteristics of the dsNSCs and their responses to a stressful aversive experience.

In mice, adult neurogenesis plays an important role in the antidepressant effects of fluoxetine (*21*). However, while fluoxetine does not enhance neurogenesis in the V-SVZ, chronic treatment increases neuron production in the DG (*18*, *21*, *22*, *88*). Previous studies indicate that fluoxetine induces hippocampal neurogenesis-dependent as well as hippocampal-independent effects, particularly in the control of anxiety (*18*). Some of the previously reported hippocampal neurogenesis-independent effects of fluoxetine may, in part, be explained by the neurogenic activity we describe here in the LSW. We show that LSW dsNSCs, like NSCs in the V-SVZ and DG, express 5HT receptors (*12*, *90*). In line with our demonstration of a fluoxetine-sensitive neurogenic niche in the mouse septum, dsNSCs could respond directly to 5HT, as SSRIs cause substantial increases in 5HT accumulation in the dorsal LSW (*74*). However, fluoxetine also has effects beyond blocking SERT and increasing 5HT levels, including modulating the activity of the tyrosine kinase receptor 2 (TRKB) (*91*). It is tempting to speculate that adult-born septal neurons are involved in some of the behavioral effects of fluoxetine and adaptations to stressful experiences. Enhanced GABAergic synaptic transmission can cause antidepressant-like behavior (*92*). Thus, the newly generated GABAergic interneurons in the LS could play anxiolytic roles in the modulation of septal circuits. Whether fluoxetine-induced local septal neurogenesis contributes to its effects on depression and anxiety remains to be determined.

Although *Notch2* deletion and fluoxetine treatment produced additive effects on progenitor proliferation in the dorsal LSW, this did not translate into an increase in GFP^+^NeuN^+^ neurons in the septum over *Notch2* cKO alone. Thus, while fluoxetine does not seem to directly affect Notch signaling in dsNSCs, it is unclear whether *Notch2* deletion and fluoxetine act through overlapping pathways. The dissociation between progenitor expansion and neuronal differentiation indicates that enhanced progenitor proliferation alone is not sufficient to drive productive neurogenesis in the dorsal LSW. Possible explanations could include limiting differentiation signals, or increased cell death of newly generated neurons, or changes in cell fate. However, our results are consistent with findings in the DG where fluoxetine enhances neurogenesis by promoting progenitor activation and proliferation rather than directly inducing neuronal differentiation (*22*). Further studies will be required to determine how newly generated septal neurons survive, mature, and integrate into local circuits.

Together, our findings identify neurogenic NSCs in the dorsal LS, in a previously unrecognized adult niche, that are responsive to aversive experiences and antidepressant treatment. However, our findings do not define the functional relevance of this neurogenic niche. Elucidating this will be important for understanding its potential role in pathophysiology, including in anxiety, stress resilience, and potentially also depression. In addition, the response of dsNSCs in the LSW to other forms of stress or insult and the longer-term mechanism regulating the integration of these newborn neurons into septal circuits remain unknown. Here, we did not address the effects of activation on the maintenance of the neurogenic capacity of the LSW, although we show that Hes5^GFP+^ putative dsNSC do not diminish with age, in contrast to what has been shown for the other adult neurogenic stem cell populations in the mouse.

## MATERIALS AND METHODS

### Experimental model and subject details

#### Animal models

The following transgenic mouse strains and alleles were used in the experiments: *Hes5::GFP* (*93*), *BLBP::mCherry* (*56*), *Hes5::CreER^T2^* (*58*), *Notch2::CreER*^*T2*-*SAT*^ (*94*), *Rosa26R::GFP* (*95*), *Rosa26R::tdTomato* (*96*), *Notch2^lox/lox^* (*97*), *Notch1^lox/lox^* (*98*), *Rbpj^lox/lox^* (*99*), and *SERT::Cre* (*100*). All mice were maintained on a C57BL/6J genetic background.

Breeding strategies for the various transgenic mouse lines were developed according to the experimental question. To confirm the expression of *Notch2*, *Notch2::CreER*^*T2*-*SAT*^ mice were bred with *Rosa26R::tdTomato* mice. To generate *Notch2*, *Notch1Notch2*, *Rbpj* cKO mice and follow *Hes5*-expressing NSCs and their progeny, *Hes5::CreER^T2^* mice were bred with *Rosa26R::GFP* and either *Notch2^lox/lox^*; *Notch1^lox/lox^Notch2^lox/lox^* or *Rbpj^lox/lox^* mice, respectively. The recombination efficiencies of the different mutants have been extensively quantified and characterized previously (*61*, *69*). Age-matched mice of both sexes were used in these studies. Mice were randomly selected for the experiments based on birth date and genotype. The *N* value in the figure legends represents the number of mice analyzed in each experiment. To predetermine sample size, we have used the Animal Experimentation Sample Size Calculator with known or estimated mean and SD values for control and experimental groups. The sample sizes were estimated to achieve a significance value in the analyses, *P* < 0.05, using an experimental prediction of Power = 90%.

All mice were bred and kept in a specific pathogen–free animal facility with 12-hour day/night cycle and free access to clean food and water according to Swiss Federal and Swiss Veterinary office regulations. All mice were healthy and immunocompetent. All procedures were approved by the Basel Cantonal Veterinary Office under license numbers 2537, 2538, 2956, 2962, and 3013 (Ethics commission Basel-Stadt, Basel Switzerland).

### Method details

#### Drug administration in mice

Age-matched mice between 8 and 10 weeks of age at the start of the experiments were used in these studies. Activation of Cre in *Notch2::CreER*^*T2*-*SAT*^ mice and *Hes5::CreER^T2^* mice carrying either floxed *Notch2* or floxed *Notch1* or floxed *Rbpj* alleles was induced by administration of tamoxifen (100 mg/kg) in corn oil for 5 consecutive days by intraperitoneal injections. Mice were euthanized, and phenotypic analyses were performed at either 2, 21, and/or 100 days after the end of the tamoxifen treatment.

Eight- to 10-week-old *Hes5::GFP; BLBP::mCherry* mice were administered fluoxetine (18 mg/kg) once daily for 7 consecutive days per os (p.o.). Vehicle-treated animals received gelatin. Mice were euthanized 1 or 19 days after the final treatment. A cohort of *Hes5::CreER^T2^* and floxed *Notch2* animals received intraperitoneal injections of tamoxifen (100 mg/kg) in corn oil for 5 consecutive days and p.o. administration of fluoxetine (18 mg/kg) or gelatin for another 7 consecutive days. Mice were euthanized 14 days after the treatment.

#### Stereotactic injection of viral particles

Adult (8- to 10-week-old) mice were administered buprenorphine (0.1 mg/kg) subcutaneously 30 min before surgery. Mice were anesthetized in a constant flow of isoflurane (1 to 3%) in oxygen and immobilized on a stereotaxic apparatus (David Kopf instruments). Bupivacaine (3 mg/kg) and lidocaine (10 mg/kg) were injected subcutaneously at multiple locations around the local area of the operation. The scalp was shaved, and the skull was exposed by an <5-mm incision in the scalp. A small hole (1 mm) was drilled through the skull. Animals were stereotactically injected with 1 μl of titrated adeno-*Gfap::Cre* virus (10^12^ infectious particles/ml) in saline containing 0.1% bovine serum albumin, using sharpened borosilicate glass capillaries (Kwick-FilTM) at the coordinates relative to bregma: anterior/posterior, 0 mm; medial/lateral, 0 mm; dorsal/ventral, 2.5 mm below the skull. Wounds were sutured closed with 5/0 Prolene suture (nonabsorbable). One day after the surgery, the animals received meloxicam (5 mg/kg) subcutaneously. Sutures were removed after wound healing and, at the latest, 10 days after surgery. Animals were analyzed 21 and 100 days poststereotactic injection.

Adult (24-week-old) male *SERT::Cre* heterozygous mice were used for labeling of dorsal raphe serotonergic neurons, using an adeno-associated virus (AAV) encoding FLEx-axon-GCaMP6s driven by the human Synapsin1 promoter. pAAV-hSynapsin1-FLEx-axon-GCaMP6s was a gift from L. Tian (Addgene viral prep #112010-AAV5; http://n2t.net/addgene:112010; RRID:Addgene 112010) (*54*). Five hundred nanoliters of the virus was injected stereotactically into the dorsal raphe of mice under anesthesia (as described above) using a stereotaxic frame (Kopf Instruments). Injections were performed at the following coordinates relative to bregma: anterior/posterior, −4.36 mm; medial/lateral, 0 mm; dorsal/ventral, 3 mm. The well-being of all mice was monitored throughout the entire experimental period.

#### Intracranial AraC infusion

AraC treatment was performed as described previously (*3*, *101*). Briefly, adult mice were anaesthetized by intraperitoneal injection of a ketamine/xylazine/flunitrazepam solution (100, 5, and 0.4 mg/kg body weight, respectively) and positioned in a stereotaxic apparatus (David Kopf Instruments, Tujunga, CA, USA). The skull was exposed by a skin incision, and a small hole (1 mm) was drilled through. AraC (2% in 0.9% saline) or vehicle alone was infused into the brain for 6 days using an osmotic pump (model 1007D; Alzet). Cannulas (Brain infusion kit II; Alzet) were implanted at the following coordinates: 0 mm anteroposterior, 1.1 mm lateral to bregma, and 0.1 mm below the surface of the cortex. After 6 days of infusion, the pump was removed, and animals were euthanized 5 days after pump removal. A single intraperitoneal injection of BrdU (50 mg/kg body weight) was administered 2 hours before sacrifice. Brains were processed and analyzed by immunohistochemistry as described below.

#### Generation of adeno-Gfap::Cre virus particles

Generation of adeno-*Gfap::Cre* virus was described previously (*71*). Briefly, Cre was placed under the control of the mouse *Gfap* promoter (GFAPp) previously confirmed to be specifically active in GFAP^+^ cells. The pAd/PLGFAPp- NLSCre-pA vector was transfected into human embryonic kidney 293 cells to produce replication-defective adenovirus, which was purified twice by cesium chloride banding. The titer was 1 × 10^12^ infectious particles/ml.

#### FC experiments

Behavioral experiments were conducted during the animal’s light period using a 4-day auditory FC paradigm. This paradigm comprised sessions in a habituation/retrieval context (days 1, 3, and 4) and an FC context (day 2). During habituation, mice were exposed to five intermingled conditioned stimuli (CS+ and CS−, 6 and 12 kHz, intermingled) in a round Plexiglass context (25 cm in diameter). Each CS consisted of 27 tone pips (200 ms, 75 dB) presented at a rate of 1.1 Hz using the Tucker Davis Technologies RZ6 system.

FC was performed in a 25-cm^2^ Plexiglass box equipped with a shock grid floor (Coulbourn, Noldus). The CS+ was paired with a 2-s, 0.65-mA foot shock delivered 1.1 s after the last tone pip. During this session, mice received five intermingled CS+ US pairings and five isolated CS− presentations. Control animals were not exposed to foot shocks. Retrieval sessions (days 3 and 4) were conducted in the habituation context, where five CS− and five CS+ stimuli were presented.

#### Administration of BrdU via drinking water

For FC-BrdU experiments, mice were exposed to sterile drinking water supplemented with BrdU (0.8 mg/ml) and 1% glucose (BrdU^+^ water). BrdU^+^ water bottles were placed in the home cage immediately following the FC session (day 2) and were removed 24 hours after the fear R2 session (day 5). Fresh BrdU^+^ water bottles were supplied daily to the mice at R1 (day 3), R2 (day 4), and day 5 of the FC experiment. Changes in BrdU^+^ water bottle weight were recorded daily to monitor fluid intake as a proxy for BrdU consumption.

#### Behavioral tracking and analysis

Behavioral tracking during the FC session was performed using EthoVision 14 (Noldus). The center of mass of each mouse was tracked, and freezing was initially detected automatically based on periods of immobility exceeding 1 s. These detections were manually reviewed and adjusted to exclude nonfreezing episodes, such as grooming.

For habituation and retrieval sessions, mouse positions were tracked using DeepLabCut (*102*). Training data consisted of 60 frames per video from five videos of three animals in varied contexts and light conditions. Mouse body parts (left and right ears, back, and base of the tail) were labeled for network training, which was performed for 200,000 iterations. Data points with a confidence level below 0.6 were excluded.

For FC-BrdU experiments, mice were tracked using contour tracking, and freezing behavior was automatically scored using frame-by-frame pixel change analysis (https://github.com/Defense-Circuits-Lab). A minimum freezing duration threshold of 1 s was applied. Automatically detected freezing events were cross-validated with video recordings to eliminate false positives and negatives, such as those caused by grooming or cable movement artifacts. Freezing data were analyzed using MATLAB (MathWorks) and Python.

#### Septum wholemount preparation and immunostaining

For septum wholemount preparations, mice were euthanized by CO_2_ exposure followed by decapitation. Brains were excised in L15 medium, and the septum was dissected under a binocular microscope. The dissected septum was fixed in 4% paraformaldehyde (PFA) solution buffered in 0.1 M phosphate buffer (PB), overnight at 4°C. After fixation, the septum was washed in 0.1 M PB, sagittally divided into two halves, and stored in 0.1 M PB supplemented with 0.02% sodium azide at 4°C until immunofluorescence processing.

For immunofluorescence staining, septum wholemounts were blocked in 20% normal donkey serum in phosphate-buffered saline (PBS) containing 2% Triton X-100 for 1 hour at room temperature. Primary antibodies were diluted in PBS supplemented with 10% normal donkey serum and 1% Triton X-100, and wholemounts were incubated for 48 hours at 4°C. The following primary antibodies were used: anti–β-catenin (rabbit, 1:1000), anti-GFP (chicken, 1:250), anti-serotonin (rabbit, 1:500), and anti-tubulin acetylated (mouse, 1:700). After extensively washing in PBS, septum wholemounts were incubated at 4°C for 24 hours with the corresponding secondary antibodies (diluted 1:600 in PBS supplemented with 10% normal donkey serum and 1% Triton X-100 solution). The secondary antibodies used were as follows: Alexa Fluor 488 donkey anti-chicken, cyanine Cy3 donkey anti-rabbit, and Alexa Fluor 647 donkey anti-mouse. Sections were counter-stained with 4′,6-diamidino-2-phenylindole (DAPI; 0.4 μg/ml) for 30 min at room temperature. Stained septum wholemounts were mounted on glass slides (Frosted, VWR), embedded in mounting medium containing diazabicyclo-octane (DABCO) as an antifading agent, and imaged using a Zeiss Axio Imager Z1 Apotome2.

#### Brain tissue preparation and immunostaining

For histological analysis, mice were deeply anaesthetized by intraperitoneal injection of a ketamine/xylazine/acepromazine mixture (130, 26, and 4 mg/kg, respectively). Anaesthetized mice were transcardially perfused with ice-cold 0.9% saline solution, followed by ice-cold 4% PFA in 0.1 M PB. Brains were isolated, postfixed overnight at 4°C in 4% PFA in 0.1 M PB, washed in 0.1 M PB, and cryoprotected in 30% sucrose in 0.1 M PB at 4°C. After 48 to 72 hours, cryoprotected brains were embedded and frozen in Tissue-Tek O.C.T. Compound. Thirty-micrometer cryostat (Leica CM3050 S)–sectioned, free floating coronal sections were collected in 24-well plates and stored at −20°C in antifreeze solution [30% glycerol, 30% ethylene glycol and 10% buffer F (pH 7.4) (190 mM sodium phosphate dibasic dihydrate) in double-distilled H_2_O] until further use.

For immunostaining, brain sections were randomly selected on the basis of anatomical location (approximately between bregma +1.3 and +0.5 mm) from different serial collecting wells (12-well format) for each individual mouse. Each well contained serial coronal sections collected from anterior to posterior with a spacing of 330 μm. Sections were blocked at room temperature for 30 min with 10% normal donkey serum in PBS containing 0.5% Triton X-100. Primary antibodies were diluted in PBS supplemented with 2% normal donkey serum and 0.5% Triton X-100, and sections were incubated overnight at 4°C. For PCNA and RBPj detection, antigen retrieval was performed by incubating sections at 80°C for 20 min in sodium citrate (10 mM, pH 6.0) before primary antibody incubation. For BrdU detection, sections were treated with 2 N HCl at 37°C for 35 min and then equilibrated in borate buffer (0.1 M, pH 8.5) for 10 min, before primary antibody incubation. The following primary antibodies were used: anti-BrdU (rat, 1:2000), anti-calbindin D28k (mouse, 1:5000), anti-calbindin D28k (rabbit, 1:5000), anti-calretinin (rabbit, 1:5000), anti-CD31 (rat, 1:500), anti-doublecortin (goat, 1:400), anti-dsRed (rabbit, 1:500), anti-GAD67 (mouse, 1:1000), anti–glial fibrillary acidic protein (mouse, 1:500), anti-Glial fibrillary acidic protein (rabbit, 1:1000), anti-GFP (chicken, 1:250), anti-GFP (rabbit, 1:500), anti-GFP, (sheep, 1:250), anti-Neuronal nuclear antigen (mouse, 1:300), anti-NOTCH2 (rat, 1:100), anti-parvalbumin (mouse, 1:5000), anti–phospho-CREB (Ser^133^) (rabbit, 1:200), anti–proliferating cell nuclear antigen (mouse, 1:1000), anti-RBPj (rabbit, 1:1000), anti-serotonin (rabbit, 1:500), and anti-somatostatin (rat, 1:500). After extensively washing in PBS, brain sections were incubated at room temperature for 2 hours with the corresponding secondary antibodies (diluted 1:600 in PBS supplemented with 10% normal donkey serum and 0.5% Triton X-100 solution). The secondary antibodies used were obtained from Jackson ImmunoResearch Ltd. Sections were counter-stained with DAPI (0.4 μg/ml) for 15 min at room temperature. Stained sections were mounted on glass slides (Frosted, VWR) and embedded in mounting medium containing DABCO as an antifading agent. Fluorescent imaging was performed using a Zeiss Axio Observer Z1 Apotome2 or a Leica SP5 confocal or Leica Stellaris 8 Falcon confocal microscopes.

#### Imaris image analysis

Orthogonal view and three-dimensional (3D) reconstruction analysis of neurons was performed using the Imaris software (version 9.3.1, Bitplane, Zurich, Switzerland). Z-stack images acquired with the Leica Stellaris 8 Falcon confocal microscope were imported into Imaris for analysis. The “Crop 3D” tool was used to isolate the region of interest containing the target neuron and focus the subsequent analysis when it was needed.

Orthogonal visualization of neurons was performed using the “Orthogonal View” function to enable precise visual confirmation of colocalization of fluorescence signals in three dimensions. Simultaneous cross-sectional views along the *XY*, *XZ*, and *YZ* planes were generated, and crosshairs were positioned to target specific points of interest. Orthogonal views were exported as high-resolution images or screen captures.

For neuronal 3D reconstruction, each fluorescence channel was rendered using the “Surfaces” module, with surface creation based on absolute intensity thresholding. Smoothing detail was set to 0.181 μm, and the minimum number of connected voxels required for surface generation was set to 10. Absolute intensity thresholds were applied as follows: channel 1 (blue), threshold 25; channel 2 (green), threshold 54.7; channel 3 (red), threshold 53. Manual object selection was performed using the pencil tool in “Edit” mode to ensure accurate segmentation. To refine visualization and signal specificity, the “Mask Selection” tool was applied. For channel 3 (red), the corresponding surface was selected, and the option “Set voxel intensity outside surface to 0” was applied, effectively removing nonsurface-associated (neuronal) signal. The resulting masked red surface appeared yellow, reflecting changes in visualization after masking. The final multichannel visualizations were generated using the “3D Blend” mode in the Surpass view, enabling integrated display of neuronal-related structures across all channels. Each channel was rendered with the following opacity and intensity settings: channel 1 (blue)—opacity 0.30, minimum 13.00, maximum 240.75; channel 2 (green)—opacity 0.03, minimum 20.18, maximum 230; channel 3 (red)—opacity 0.30, minimum 50.77, maximum 220. 3D renderings were exported as high-resolution images.

#### Ex vivo FACS

Adult (8- to 14-week-old) *Hes5::GFP* animals were euthanized by CO_2_ exposure and then decapitated. Brains were excised into Leibovitz’s L15 medium, and the septum was carefully dissected under a binocular microscope. The anterior dorsal part of the septum was isolated from 10 mice, and the pooled tissue was enzymatically dissociated in papain/trypsin inhibitor solutions mix (1:1 ratio) [papain solution: papain (30 U/ml), deoxyribonuclease I (DNase I; 40 μg/ml), and l-cysteine (0.24 mg/ml) in Leibovitz’s L15 GlutaMAX-supplemented medium; trypsin inhibitor solution: trypsin inhibitor (1.125 mg/ml), bovine serum albumin (0.525 mg/ml), and DNase I (40 μg/ml) in Leibovitz’s L15 GlutaMAX-supplemented medium] for 10 min at 37°C, followed by incubation in trypsin inhibitor solution with vigorous mechanical dissociation, and cells were collected by centrifugation at 80*g* for 5 min. The sample was washed twice in L15 medium without phenol red, and cells were resuspended in L15 medium without phenol red supplemented with 5 mM EDTA. Live cells were discriminated by forward- and side-scatter and by DAPI (0.1 μg/ml) exclusion. GFP^+^ cells were gated and sorted with a FACS AriaIII (BD Biosciences). Analysis of flow cytometry data was performed with FlowJo software.

#### RNA isolation and reverse transcriptase PCR

Total RNA was isolated from FACS-sorted Hes5^GFP+^ septal NSCs, as well as from cortex and striatum, using TRIzol reagent (Invitrogen), according to the manufacturer’s instructions with some modifications. Samples were homogenized in 1 ml of TRIzol by mechanical dissociation, followed by 5 min of incubation at room temperature. For phase separation, chloroform was added (20% of the TRIzol total volume), and the samples were centrifuged at 12,000*g* for 30 min at 4°C. The aqueous phase was collected, and the RNA precipitated overnight at −20°C with isopropanol (50% of the TRIzol total volume) and 15 μg of GlycoBlue. The RNA pellet was washed with ice-cold 75% ethanol (100% of the TRIzol total volume), air-dried, and resuspended in ribonuclease-free Milli-Q water. Reverse transcription of 10 ng of total RNA was performed using SuperScript III First-Strand Synthesis System (Invitrogen), according to the manufacturer’s instructions.

The newly synthesized cDNA was polymerase chain reaction (PCR) amplified using primer pairs for *5Htr1a*, *5Htr1b*, *5Htr1d*, *5Htr1f*, *5Htr2a*, *5Htr2b*, *5Htr2c*, *5Htr4*, *5Htr5a*, *5Htr5b*, *5Htr6*, *5Htr7*, *5Htr3a*, *5Htr3b*, and *Actb*.

PCR reactions were performed in a total volume of 20 μl, containing 0.3 μM each of forward and reverse primer by using FIREPol Master Mix (12.5 mM MgCl_2_; Solis BioDyne) according to the manufacturer’s instructions. The conditions of PCR reactions were as follows: 40 cycles of 95°C for 1 min, annealing for 1 min (see below annealing temperature for each pair of primers) and 72°C for 1 min with an initial denaturation cycle of 95°C for 5 min and a final extension cycle of 72°C for 4 min. The annealing temperature used for *5Htr1a*, *5Htr1b*, *5Htr1d*, *5Htr1f*, *5Htr2a*, *5Htr2c*, *5Htr4*, *5Htr5a*, *5Htr5b*, *5Htr6*, *5Htr7*, and *5Htr3a* was 56°C; for *5Htr2b* was 55°C; for *5Htr3b* was 57°C and for *Actb* was 60°C. Fractionation of PCR products was performed by electrophoresis in 2% agarose/tris-acetate-EDT gel containing ethidium bromide.

#### Neuronicheproteome website

Neuronicheproteome website (https://pawelsm.github.io/neuronichen1/profiles.html) is an online platform of published data (*55*) for comprehensive characterization of the neurogenic niche proteome compared to normal brain parenchyma. It was used to identify components of the 5HT biosynthesis pathway as well as NSC- and neurogenesis-associated proteins in the LSW (*55*).

#### Quantification and statistical analysis

Fiji (ImageJ, National Institutes of Health, MD, USA) software was used for quantification analysis of immunofluorescence images. Data are presented as average percentage of colabeled cells or as average cell density per unit volume (cells per cubic millimeter) and as average cell density per sectional area (cells per square millimeter; shown in the supplementary figures). A minimum of three to four sections were analyzed per animal, between bregma +1.3 and +0.5 mm, with the sample size (*N*) for each experiment indicated in the figure legends. The dorsal LSW and the quantified ventricular zone regions were defined using the anatomical coordinates and boundaries from the Franklin and Paxinos atlas (*103*). The dorsal boundary of the septum was defined as the corpus callosum and the lateral boundary as the ventricular surface. The ventral boundary of the dorsal septal domain was defined as the ventral limit of the dorsal lateral septal nuclei. This approximates to the dorsal third of the ventricular length from the ventricular surface beneath the corpus callosum. The septal ventricular zone for quantifications (of PCNA^+^, DCX^+^, and BrdU^+^ cells) was defined as extending 50 μm from the septal wall into the septal parenchyma. NeuN^+^ neurons were quantified throughout the entire septum from the corpus callosum to the ventral limit of the ventral LS nuclei. The V-SVZ of the lateral ventricular wall was excluded from all calculations.

Statistical significance was determined by two-tailed unpaired Student’s *t* test on mean values per animal using GraphPad Prism v9 (GraphPad Software Inc., La Jolla, CA, USA). Statistical analysis of percentages was performed following the transformation of percentages into their arcsine square root value. The *t* values (*t*) and df for *t* tests are indicated in the figure legends. For experiments with *N* = 3 animals per group (Figs. 1, C and D, and 3G), we report both *P* values and effect sizes (Cohen’s *d*) for all comparisons to provide a more complete assessment of the data. We note that the small sample sizes of *N* = 3 per group may limit statistical power and may inflate effect size estimates.

For FC experiments, differences in the percentage of freezing within and between FC and Ctrl groups were assessed using the Wilcoxon signed-rank test. The relationship between freezing behavior and molecular markers of neurogenesis was assessed using Spearman’s correlation. For each animal, the percentage of freezing during conditioning (end FC), each of the two retrieval sessions alone (R1 or R2), as well as the average of the two retrieval sessions together (R1 and R2) was plotted against the expression of the molecular marker of interest. Spearman’s correlation coefficient (*r*) and associated *P* values were used to evaluate the strength and significance of correlations.

Significance was determined at *P < 0.05, **P < 0.01, and ***P < 0.001, or *P* values are given in the graphs. Deviance from mean is displayed as SD if not otherwise indicated.

## Supplementary Materials

### The PDF file includes:

Figs. S1 to S10 Table S1 References
