# Supplementary material for: Mice produce interneurons in the septum as a response to aversive experiences and antidepressant treatment
Source: Sci Adv. 2026 Jun 10;12(24):eaed3625. doi: 10.1126/sciadv.aed3625 (PMC13251870; doi:10.1126/sciadv.aed3625)
Supplement: Supplementary file 1 — Figs. S1 to S10 Table S1 References [file sciadv.aed3625_sm.pdf]

Science Advances
AAAS

Supplementary Materials for

Mice produce interneurons in the septum as a response to aversive experiences and antidepressant treatment

Aikaterini Lampada *et al.*

Corresponding author: Verdon Taylor, verdon.taylor@unibas.ch

Sci. Adv. **12**, eaed3625 (2026)
DOI: 10.1126/sciadv.aed3625

The PDF file includes:

Figs. S1 to S10
Table S1
References

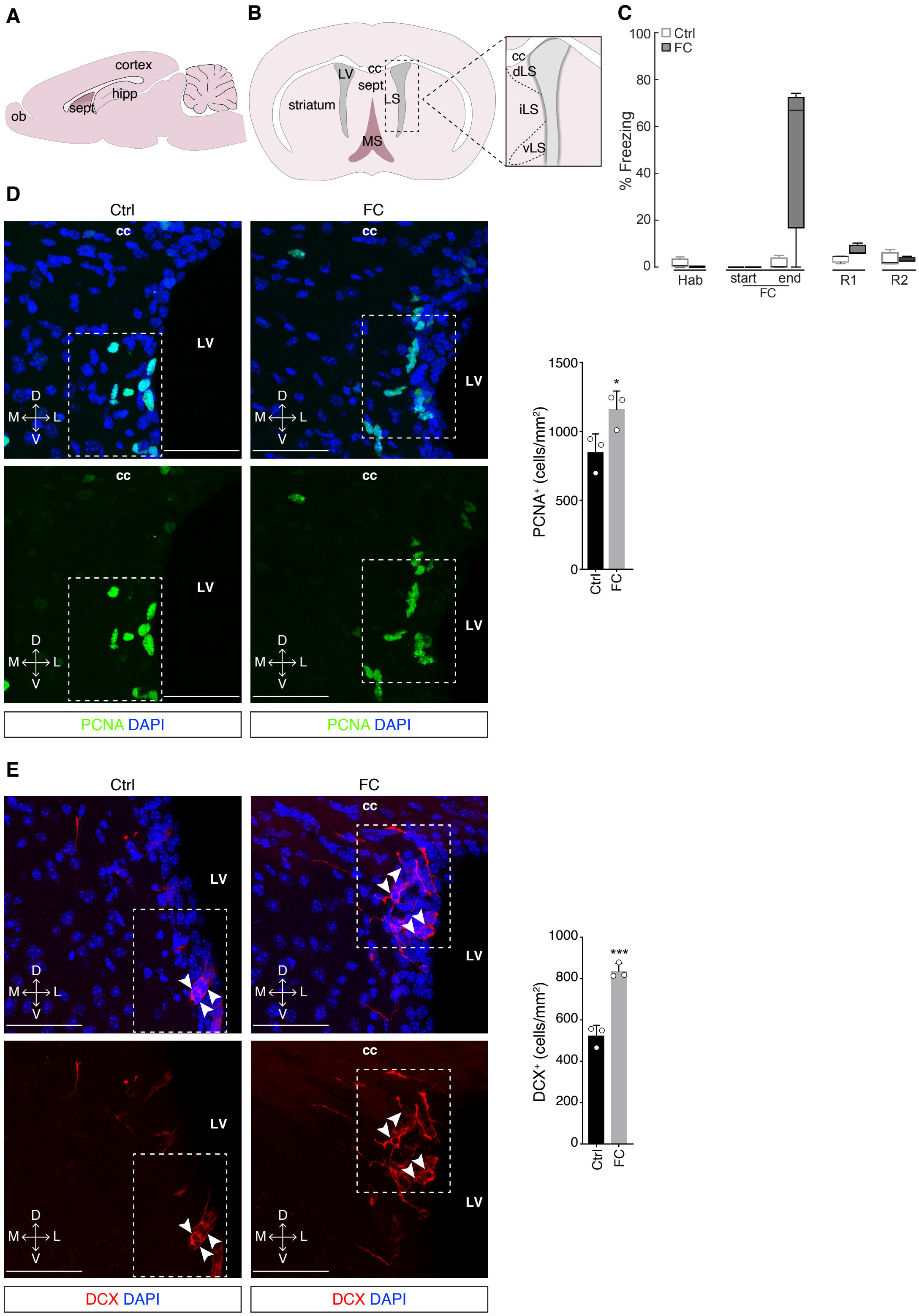

Fig. S1. FC induces neurogenesis in the dorsal lateral septum.(**A**) Scheme of a sagittal section of the adult mouse brain, showing the location of the septum. The septum lays dorsomedial to the hypothalamus and pallium in the subcortical forebrain under the corpus callosum and anterior to the dorsal hippocampus. (**B**) Coronal view of the septum in the adult mouse brain, showing the lateral and medial septal (LS, MS) subregions. Dotted rectangle marks the LS and corresponds to the magnified projection of the LS with its dorsal, intermediate and ventral subdivisions (dLS, iLS, and vLS). (**C**) Box plot depicting freezing behavior of Ctrl (N=3) and FC (N=3) mice in

response to the conditioned stimulus (CS+) across all experimental phases of the paradigm. The percentage (%) of freezing was measured during the habituation (Hab), FC phases (start and end), retrieval 1 (R1) and retrieval 2 (R2). Data are presented as the median (line), interquartile range (box), and full data range (whiskers). (**D**-**E**) Images showing a low-magnification view of the dorsal LSW of Ctrl and FC mice and quantifications of cell densities per sectional area (cells / mm^2), 1 day after the completion of the FC paradigm. Panels and corresponding bar plots in D and E show $PCNA^+$ cells (green) and DCX^+ neuroblasts (red, arrowheads in E), respectively. Dotted rectangle marks the magnified region of interest in Fig. 1C and D. Bar plots show cell densities per sectional area corresponding to the volumetric densities presented in Fig. 1C and D. $PCNA^+$ and DCX^+ cells were quantified in the dorsal LSW of both hemispheres on 3-4 coronal sections per brain. Bars represent means ± SD of N=3 Ctrl, and N=3 FC. Statistical significance was calculated by an unpaired t-test: *P<0.05, ***P<0.001. Scale bars: 50 µm in D and E. cc, corpus callosum; sept, septum; hipp, hippocampus; ob, olfactory bulb; LV, lateral ventricle; dLS, dorsal lateral septum; iLS, intermediate lateral septum; vLS, ventral lateral septum; D, dorsal; V, ventral; M, medial and L, lateral orientation of the mouse brain.

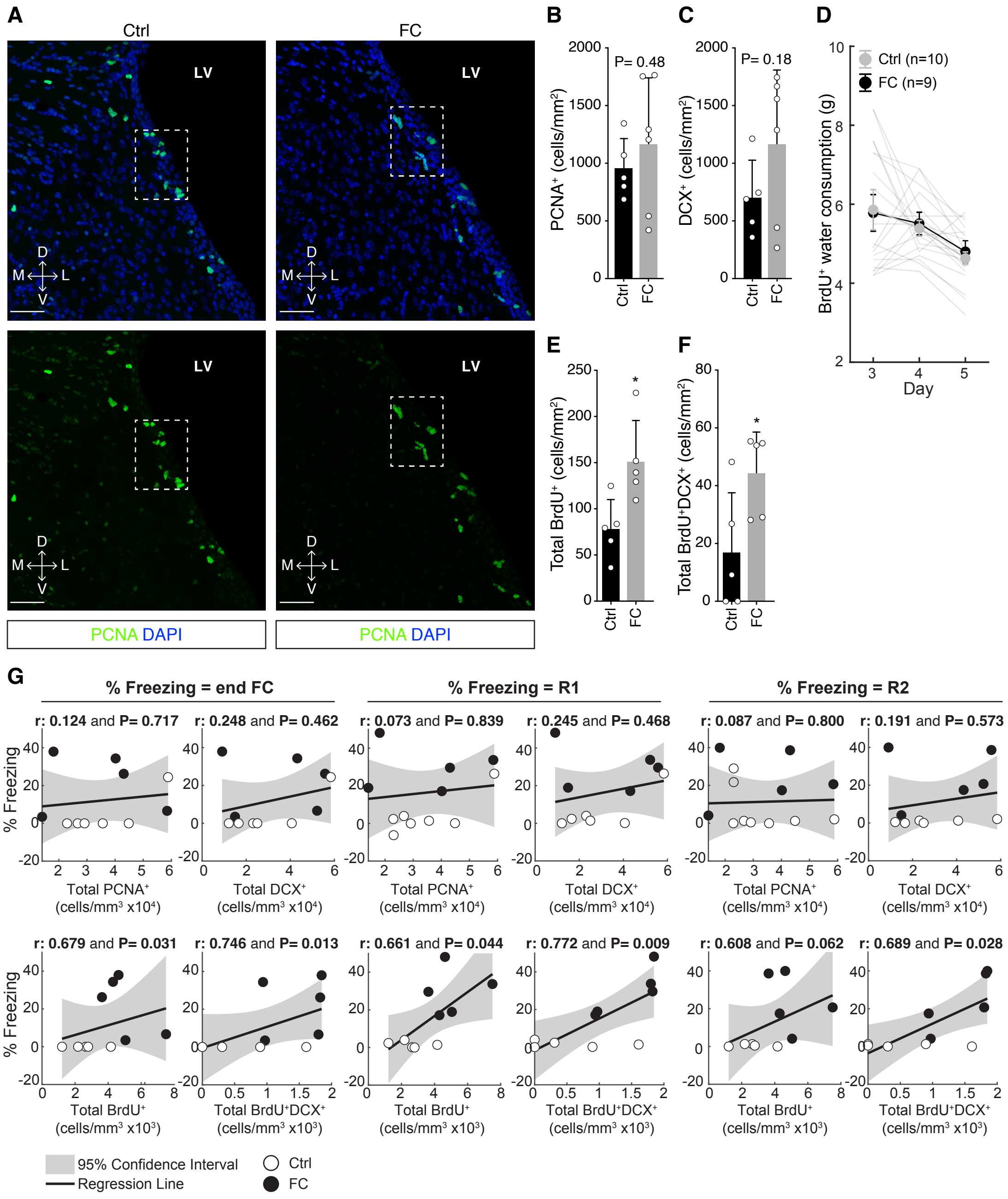

Fig. S2. Neurogenesis is an acute response to FC. (**A**) Images showing a low magnification view of the dorsal LS of Ctrl and FC mice, 18 days after the completion of the FC-BrdU paradigm. Panels show PCNA^{+} cells (green). Dotted rectangle marks the magnified region of interest in Fig. 1F. Scale bars: 50 µm. (**B**) Quantification of PCNA^{+} cells in the dorsal LSW of Ctrl and FC mice, 18 days after the completion of the FC-BrdU paradigm. Bar plot shows cell densities per sectional area corresponding to the volumetric densities presented in Fig. 1F. PCNA^{+} cells were quantified in the dorsal LSW of both hemispheres on 3 coronal sections per brain. Bars represent means ± SD of N=5 Ctrl, and N=6 FC. Statistical significance was calculated by an unpaired t-test. (**C**) Quantification of DCX^{+} neuroblasts in the dorsal LSW of Ctrl and FC mice, 18 days after the completion of the FC-BrdU paradigm. Bar plot shows cell densities per sectional area corresponding to the volumetric densities presented in Fig. 1G. DCX^{+} cells were quantified in the dorsal LSW of both hemispheres on 3 coronal sections per brain. Bars represent means ± SD of N=5 Ctrl, and N=6 FC. Statistical significance was calculated by an unpaired t-test. (**D**)

Graph depicts $BrdU^+$ water consumption of Ctrl (N=10) and FC (N=9) mice, measured as changes in water bottle weight. No difference in the consumption of $BrdU^+$ water was observed between the Ctrl and FC groups. Data are presented as means ± SEM, and individual mice are displayed (lines). Statistical significance was calculated by a repeated measures ANOVA (group x day, $F(1, 17) = 0.123$, $P=0.730$. (**E**-**F**) Quantification of $BrdU^+$ (E) and $BrdU^+DCX^+$ (F) cells in the dorsal LSW of Ctrl and FC mice, 18 days after the completion of the FC-BrdU paradigm. Bar plots show cell densities per sectional area corresponding to the volumetric densities presented in Fig. 1I and J. $BrdU^+$ and $BrdU^+DCX^+$ cells were quantified in the dorsal LSW of both hemispheres on 2-3 coronal sections per brain. Bars represent means ± SD of N=5 Ctrl, and N=5 FC. Statistical significance was calculated by an unpaired t-test: $^*P<0.05$. (**G**) Scatter plots depicting correlation analyses between neurogenesis molecular markers and freezing behavior during FC (end), retrieval 1 (R1) or retrieval 2 (R2) in Ctrl (open circles) and FC (filled circles) mice, 18 days after the completion of the FC-BrdU paradigm. Total $PCNA^+$ cells / mm^3 and total DCX^+ cells / mm^3, N=6 Ctrl, and N=5 FC; Total $BrdU^+$ cells / mm^3 and total $BrdU^+DCX^+$ cells / mm^3, N=5 Ctrl, and N=5 FC. Spearman's correlation coefficient (r) and associated P values (P) are shown. Solid lines represent the linear correlation, with shaded areas indicating the 95% confidence interval.

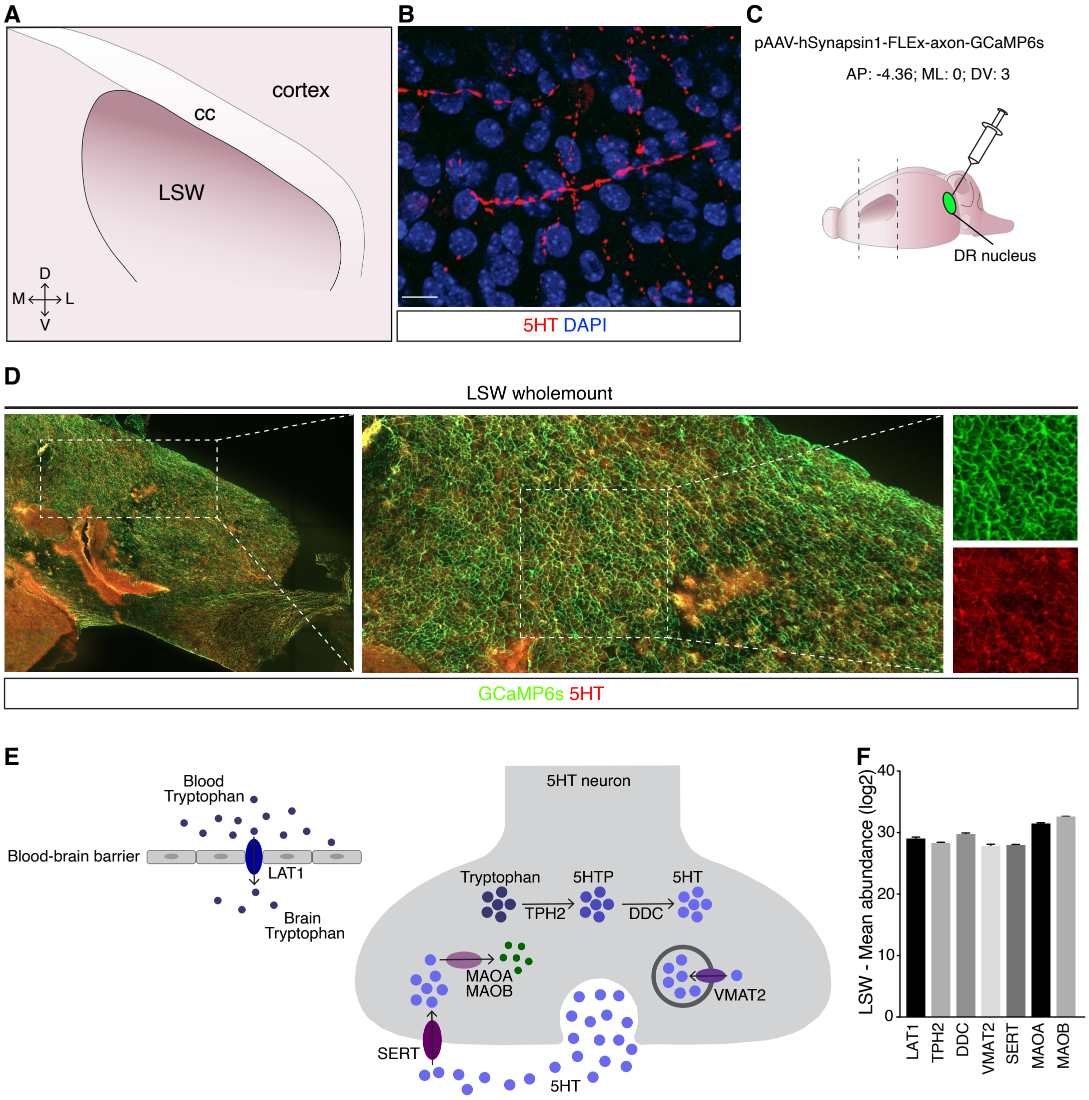

Fig. S3. 5HT signaling components in the LSW. (**A**) Scheme of a sagittal view of the adult mouse septum used for septum wholemount preparation and analysis. (**B**) *En face* image of a septum wholemount showing $5HT^+$ processes on the ependymal surface of the LSW. (**C**) Scheme depicting the coordinates of the dorsal raphe (DR) nucleus stereotactic injection procedure for 5HT neuronal-labeling using an pAAV-hSynapsin1-FLEx-axon-GCaMP6s viral construct (anterior-posterior; AP, medial-lateral; ML, and dorsal-ventral; DV). (**D**) Low magnification view of a septum wholemount showing the distribution of dorsal raphe-efferents, $GCaMP6s^+$-labelled and $5HT^+$ (red) axons, covering the ependymal surface of the LSW. Dotted rectangle and square mark the magnified images. (**E**) Scheme of the 5HT biosynthesis pathway. Tryptophan crosses the blood-brain barrier through the large neutral amino acid transporter (LAT1). Neurons that express the tryptophan 5-hydroxylase 2 (TPH2) enzyme convert tryptophan into 5-hydroxytryptophan (5HTP), which is subsequently converted into 5HT by aromatic l-amino-acid decarboxylase (DDC). Newly synthesized 5HT is transported into synaptic vesicles by vesicular monoamine transporter 2 (VMAT2). Extracellular free 5HT in the brain can be transported by the 5HT reuptake transporter (SERT) back into neurons, where it is either packaged into synaptic vesicles or catabolized by monoamine oxidase A (MAOA) and B (MAOB) enzymes. (**F**) Bar plot showing the abundance of 5HT biosynthesis pathway components in the LSW quantified by

proteomic analysis (*55*). Bars represent mean ± SD. cc, corpus callosum; LSW, lateral septum wall; D, dorsal; V, ventral; M, medial; and L, lateral orientation of the mouse brain.

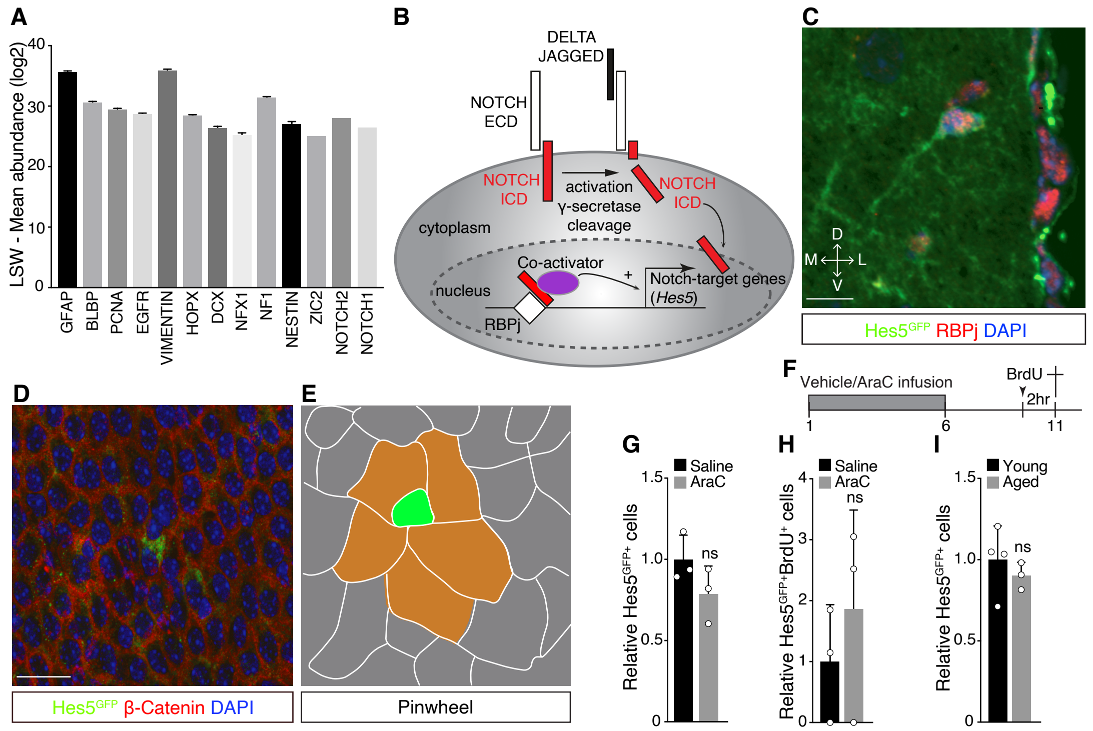

Fig. S4. Neurogenesis associated markers and Notch signaling in the LS. (**A**) Bar plot showing the abundance of NSC- and neurogenesis-associated proteins in the LSW quantified by proteomic analysis (*55*). Bars represent mean ± SD. (**B**) Schematic illustration of the Notch signaling cascade. Upon binding of a Notch ligand (a DELTA or JAGGED), the Notch intracellular domain (Notch ICD) is cleaved by γ-secretase and released from the cell membrane. Notch ICD translocates into the nucleus and interacts with the Notch signaling transcriptional regulator CSL protein, RBPj in mice, to regulate the expression of Notch-target genes (including *Hes5*). (**C**) Radial Hes5^{GFP+} cells in the dorsal LSW expressing RBPj. Scale bar: 10 µm. D, dorsal; V, ventral; M, medial and L, lateral orientation of the mouse brain. (**D**) Image of a septum wholemount preparation showing a Hes5^{GFP+} cell protruding through the LS ependyma. Ependymal adherence junctions are labelled by anti-β-Catenin immunostaining. Scale bars: 15 µm. (**E**) Scheme of a pinwheel structure formed by ependymal cells (orange) in the dorsal LSW around Hes5^{GFP+} cell protrusions (green). (**F**) Schematic representation of AraC treatment and analysis timeline, including BrdU intraperitoneal (i.p.) injection. (**G**) Bar plot showing the ratio of Hes5^{GFP+} cells in the dorsal LSW of AraC-treated mice relative to saline-treated mice. Bars represent mean ± SD of N=3 mice. Statistical significance was calculated by an unpaired t-test: ns, not significant (t=1.612 and df=4). (**H**) Bar plot of the ratio of Hes5^{GFP+}BrdU^{+} dividing cells in the dorsal LSW of AraC-treated mice relative to saline-treated mice. Bars represent mean ± SD of N=3 mice for both saline and AraC-treated. Statistical significance was calculated by an unpaired t-test: ns, not significant (t=0.4359 and df=4). (**I**) Bar plot showing the ratio of Hes5^{GFP+} cells in the dorsal LSW of aged (52-week-old) mice relative to young (8-week-old) mice. Bars represent mean ± SD of N=4 for young and N=3 for aged mice. Statistical significance was calculated by an unpaired t-test: ns, not significant (t=0.6947 and df=5).

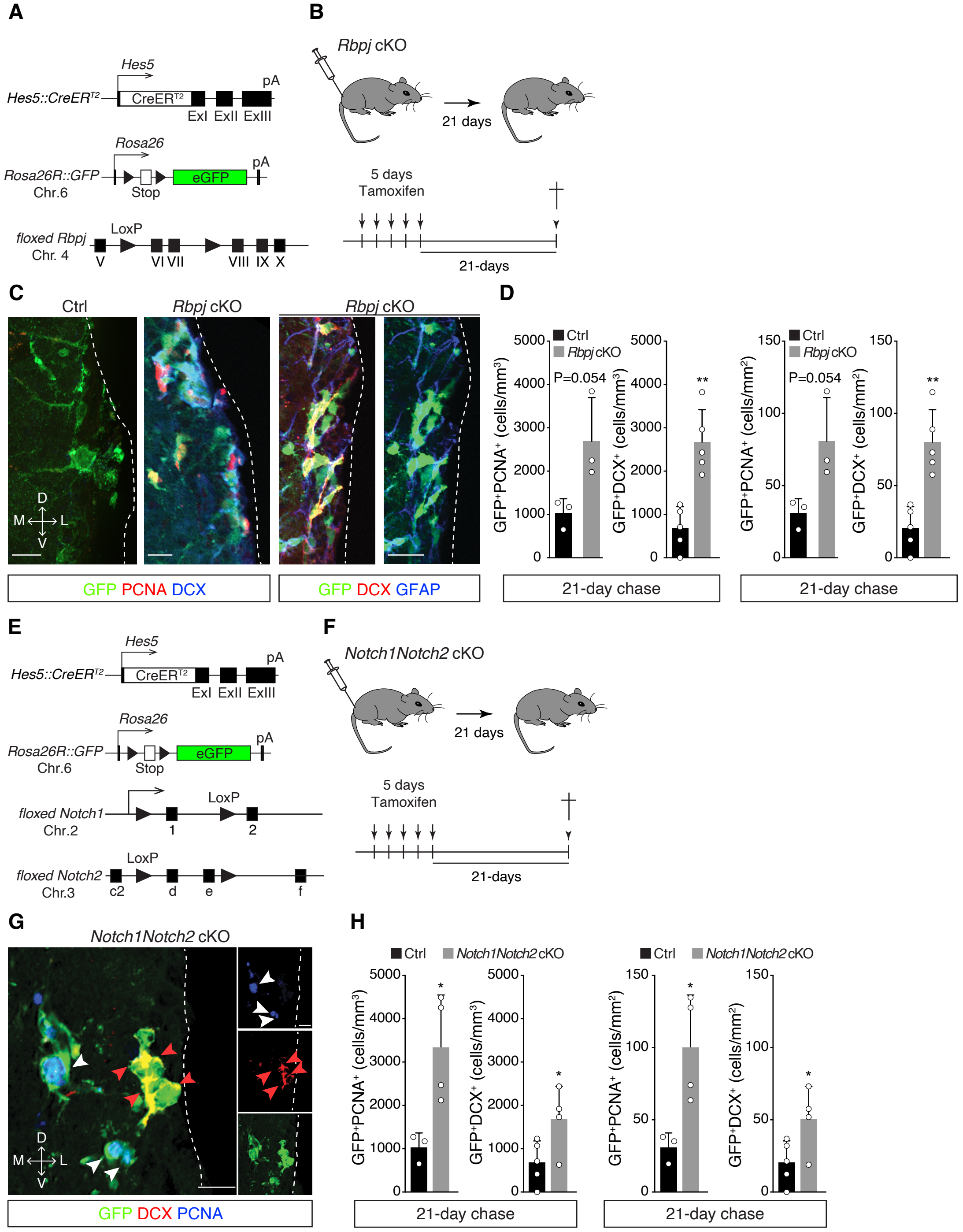

Fig. S5. *Rbpj* deletion as well as concomitant *Notch1* and *Notch2* deletion induces neurogenesis in the dorsal LSW. (**A**) Schemes of floxed *Rbpj*, *Hes5::CreER^{T2}* transgene and *Rosa26R::GFP* Cre-reporter allele, with chromosome (Chr.), exons (Ex), LoxP, and poly-adenylation (pA) sites. (**B**) Scheme of Tamoxifen administration via intraperitoneal (i.p.) injection and timeline for lineage analysis. (**C**) Image depicting *Hes5::CreER^{T2}*-derived (GFP$^+$) PCNA$^+$ and DCX$^+$ cells in the dorsal LSW of Ctrl and *Rbpj* cKO animals, 21 days after Tamoxifen administration. Dotted lines mark the LSW. Scale bar: 10 µm. D, dorsal; V, ventral; M, medial and L, lateral orientation of the mouse brain. (**D**) Quantification of *Hes5::CreER^{T2}*-derived (GFP$^+$) mitotic cells (GFP$^+$PCNA$^+$) and neuroblasts (GFP$^+$DCX$^+$) in the dorsal LSW of *Rbpj* cKO

and Ctrl animals, 21 days after Tamoxifen administration. Bar plots are presented as volumetric densities (cells / mm^3) and as cell densities per sectional area (cells / mm^2). Bars represent mean ± SD. GFP^+PCNA^+ quantification, N=3; GFP^+DCX^+ quantification, N=5 for both Ctrl and *Rbpj* cKO. Statistical significance was calculated by an unpaired t-test: **$P<0.01$, (GFP^+PCNA^+: t=2.709 and df=4; GFP^+DCX^+: t=4.925 and df=8). (**E**) Schemes of floxed *Notch1*, floxed *Notch2*, *Hes5::CreER^{T2}* transgene and *Rosa26R::GFP* Cre-reporter alleles with chromosome (Chr.), exons (Ex), LoxP, and poly-adenylation (pA) sites. (**F**) Scheme of Tamoxifen administration via intraperitoneal (i.p.) injection and lineage analysis timeline. (**G**) Image of *Hes5::CreER^{T2}*-derived $PCNA^+$ (white arrowheads) and DCX^+ (red: arrowheads) cells in the dorsal LSW of *Notch1*, *Notch2* double cKO animals. Scale bar: 10 µm. D, dorsal; V, ventral; M, medial and L, lateral orientation of the mouse brain. (**H**) Quantification of GFP^+PCNA^+ and GFP^+DCX^+ cells in the dorsal LSW of Ctrl, and *Notch1*, *Notch2* double cKO animals. Bar plots are presented as volumetric densities (cells / mm^3) and as cell densities per sectional area (cells / mm^2). Bars represent mean ± SD. GFP^+PCNA^+ quantification, N=3 Ctrl, and N=4 *Notch1*, *Notch2* cKO animals; GFP^+DCX^+ quantification, N=5 Ctrl, and N=4 *Notch1*, *Notch2* cKO animals. Statistical significance was calculated by an unpaired t-test: *$P<0.05$, (GFP^+PCNA^+: t=3.128 and df=5; GFP^+DCX^+: t=2.376 and df=7).

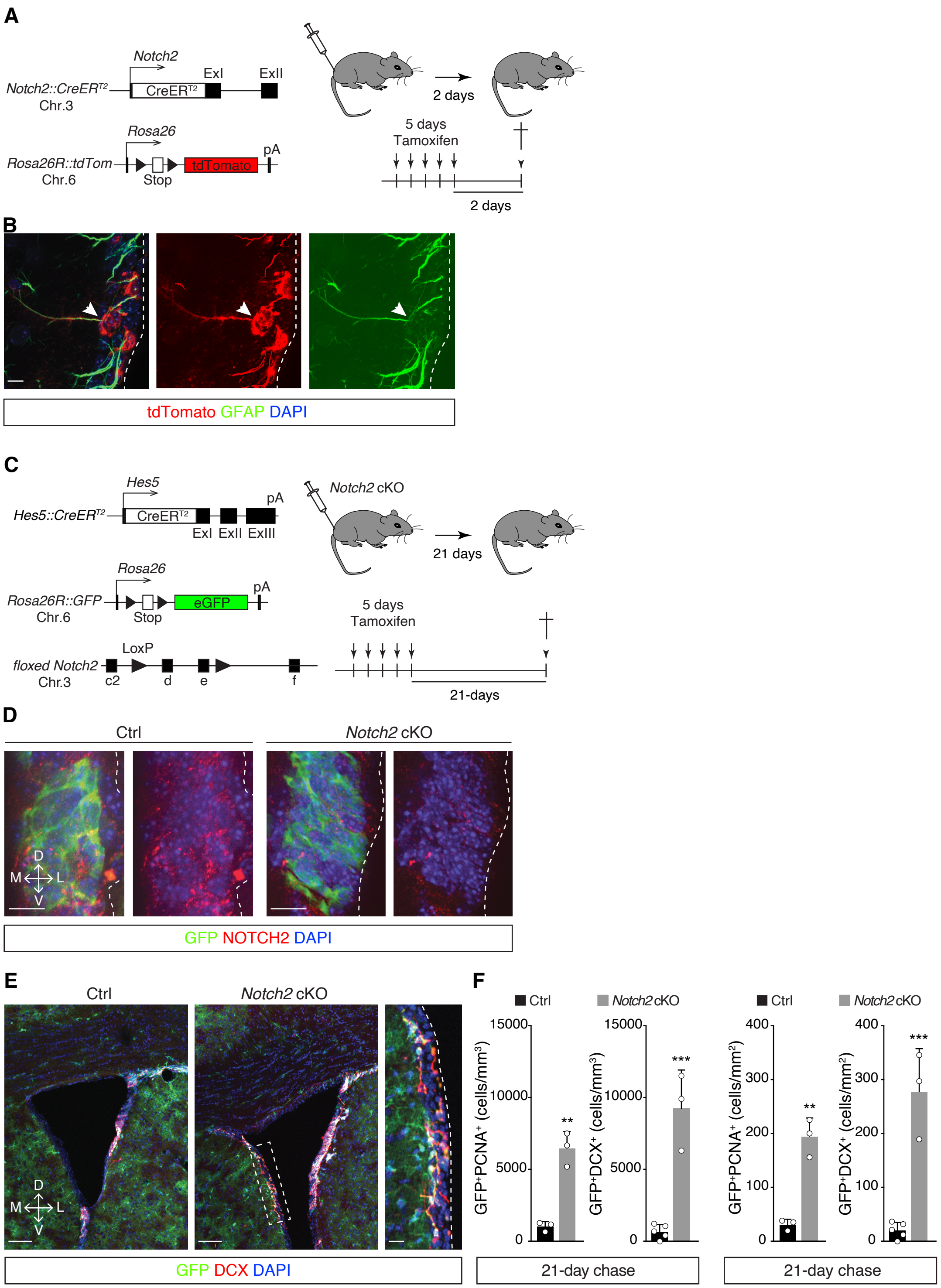

Fig. S6. *Notch2* deletion induces neurogenesis in the dorsal LSW. (**A**) Scheme of *Notch2-CreER$^{T2\text{-}SAT}$* transgene and *Rosa26R::tdTomato* Cre-reporter allele with chromosome (Chr.), exons (Ex) and poly-adenylation (pA) sites. Scheme of Tamoxifen administration via intraperitoneal (i.p.) injection and analysis timeline. (**B**) Tamoxifen-induced genetic labeling (*Rosa26R-tdTomato*) of Notch2^{+} radial GFAP^{+} cells (arrowhead) in the dorsal LSW of *Notch2::CreER$^{T2\text{-}SAT}$* animals. Scale bars: 10 µm. (**C**) Schemes of floxed *Notch2*, *Hes5::CreER^{T2}* transgene and *Rosa26R::GFP* Cre-reporter alleles with chromosome (Chr.), exons (Ex), LoxP, and poly-adenylation (pA) sites. Scheme of Tamoxifen administration via intraperitoneal (i.p.) injection and lineage analysis timeline. (**D**) Images showing the expression of NOTCH2 in

Hes5::CreER^{T2}-derived cells (GFP^{+}) cells in the dorsal LSW of Ctrl and *Notch2* cKO mice. Dotted lines mark the LSW. Scale bars: 15 µm. (**E**) Images showing *Hes5::CreER^{T2}*-derived GFP^{+}DCX^{+} neuroblasts in the dorsal LSW of Ctrl and *Notch2* cKO animals, 21 days after Tamoxifen administration. Dotted rectangle in the *Notch2* cKO image indicates the region shown in a high magnification view. Scale bars: 100 µm and 20 µm in high magnification view. (**F**) Quantification of GFP^{+}PCNA^{+} cells and GFP^{+}DCX^{+} neuroblasts in the dorsal LSW of *Notch2* cKO and Ctrl animals, 21 days after Tamoxifen administration. Bar plots are presented as volumetric densities (cells / mm^{3}) and as cell densities per sectional area (cells / mm^{2}). Bars represent mean ± SD. GFP^{+}PCNA^{+} quantification, N=3 for both Ctrl and *Notch2* cKO; GFP^{+}DCX^{+} quantification, N=5 Ctrl, and N=3, *Notch2* cKO. Statistical significance was calculated by an unpaired t-test: **P<0.01, ***P<0.001 (GFP^{+}PCNA^{+}: t=7.733 and df=4; GFP^{+}DCX^{+}: t=7.359 and df=6). D, dorsal; V, ventral; M, medial and L, lateral orientation of the mouse brain.

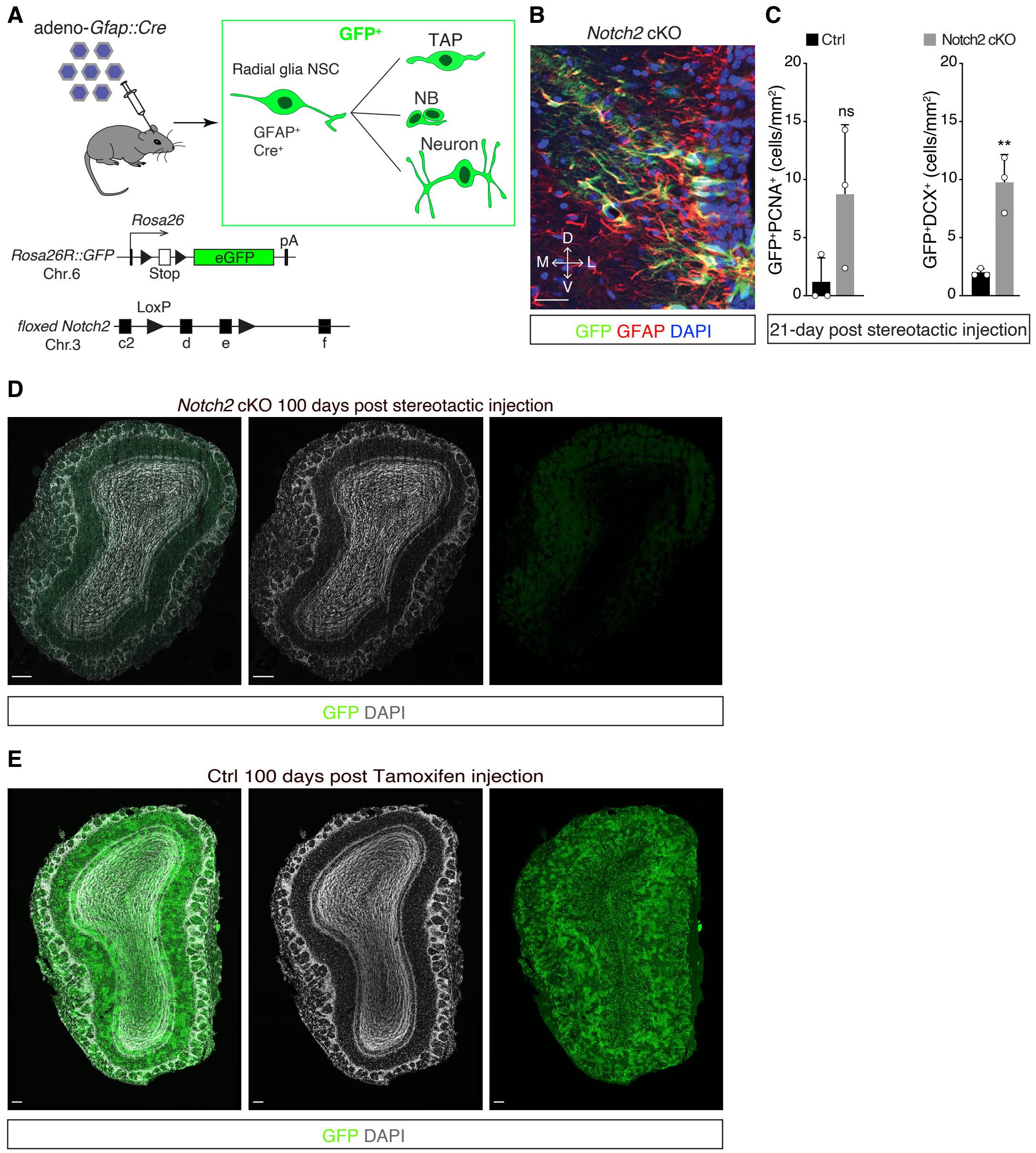

Fig. S7. Newborn septal neurons do not contribute to the generation of OB interneurons. (**A**) Scheme illustrating adeno-*Gfap::Cre*-mediated recombination of radial glia NSCs in the dorsal LS. Following stereotactic injection of adeno-*Gfap::Cre* virus into the dorsal LS of *Rosa26R::GFP* or floxed *Notch2 Rosa26R::GFP* mice, local $GFAP^+$ radial glia NSCs and their progeny express GFP enabling lineage tracing. (**B**) Image showing GFP and GFAP co-expression in the LSW following stereotactic injection of adeno-*Gfap::Cre* virus into the dorsal LS. (**C**) Quantification of GFP^+PCNA^+ cells and GFP^+DCX^+ neuroblasts derived from adeno-*Gfap::Cre* infected NSCs in the dorsal LSW of Ctrl and *Notch2* cKO animals, 21 days post-stereotactic injection. Bar plots show cell densities per sectional area corresponding to the volumetric densities presented in Fig. 3G. Bars represent mean ± SD of N=3 for both Ctrl and *Notch2* cKO. Statistical significance was calculated by an unpaired t-test: ns, not significant; $^{**}P<0.01$. (**D**) Low magnification view of the olfactory bulb (OB) of a *Notch2* cKO mouse, 100 days after stereotactic injection of adeno-*Gfap::Cre* virus in the dorsal LS. (**E**) Low magnification view of the OB of a *Hes5::CreER*^{T2} mouse, 100 days after intraperitoneal (i.p.) injection of Tamoxifen. Scale bars: 15 µm in B; 100 µm in C and D. TAP, transient amplifying progenitor cells; NB, neuroblasts; D, dorsal; V, ventral; M, medial and L, lateral orientation of the mouse brain.

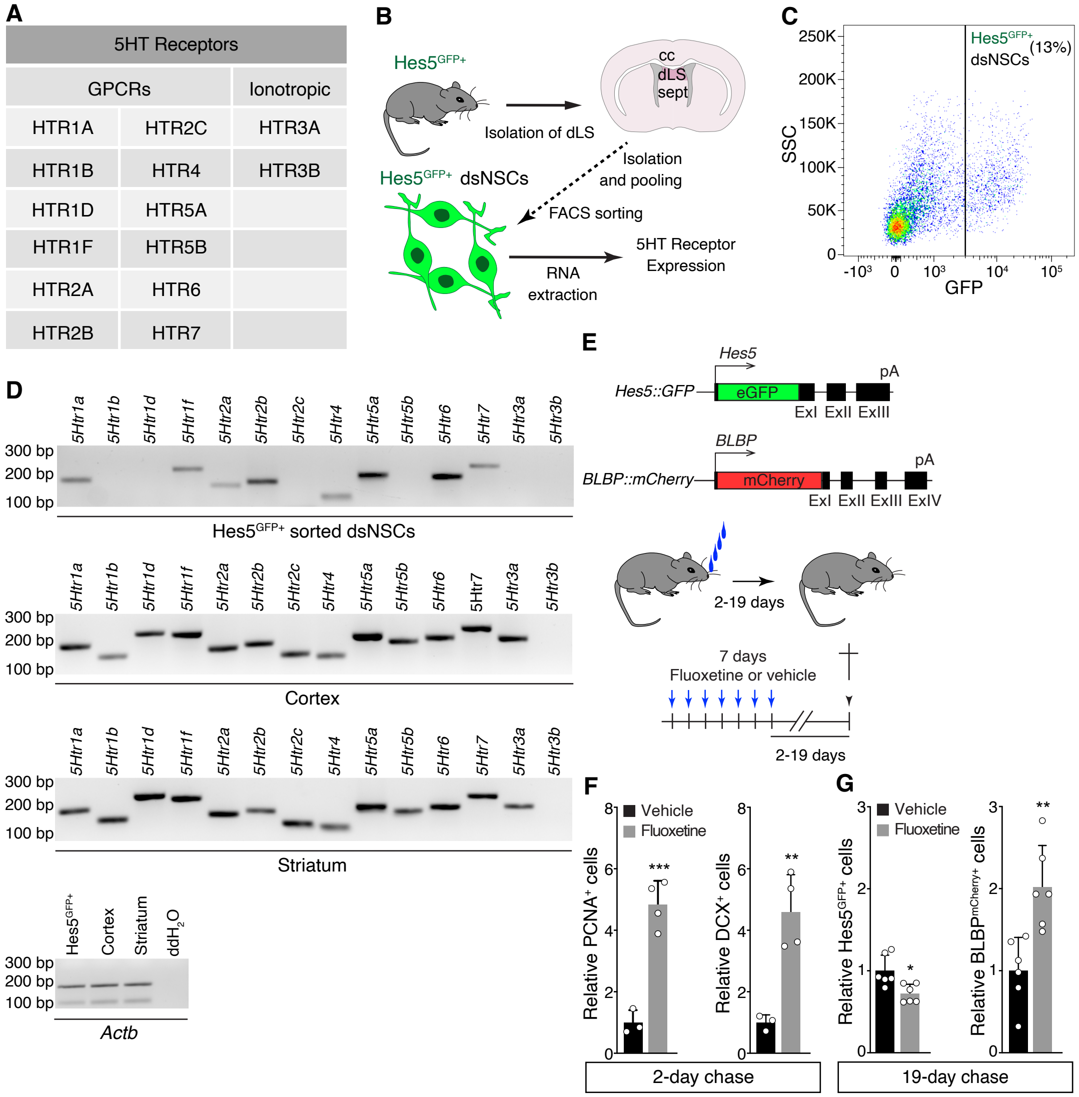

Fig. S8. Characterization of septal NSCs and activation of neurogenesis in response to Fluoxetine. (**A**) Table summarizing the 14 different 5HT receptor subtypes, both G protein-coupled receptors (GPCRs) and ionotropic receptors. (**B**) Scheme depicting the experimental procedure of septum NSC isolation for the analysis of 5HT receptor expression. The dorsal part of the septum was isolated from adult *Hes5::GFP* mice by micro-dissection. Hes5^{GFP+} dsNSCs were isolated by FACS, gating on the GFP^{+} cells. RNA was extracted from the sorted cells and 5HT receptor expression analysis was performed by RT-PCR. (**C**) Cell gating for live cells (side scatter; SSC) and isolation of Hes5^{GFP+} dsNSCs. (**D**) Gel electrophoresis images of PCR amplicons showing the expression of 14 different 5HT receptor subtypes by Hes5^{GFP+} sorted septal NSCs (dsNSCs), and cortex and striatum as controls. cDNA levels were standardized by β-actin (Actb) amplification (**E**) Schemes of *Hes5::GFP* and *BLBP::mCherry* transgenes with exons (Ex) and poly-adenylation (pA) sites. Schematic representation of vehicle or Fluoxetine administration together with the analysis timeline. *Hes5::GFP*, *BLBP::mCherry* mice received Fluoxetine or vehicle for 7 consecutive days via oral gavage. Analyses were performed 2 or 19 days after vehicle or Fluoxetine administration. (**F**) Bar plot showing the ratio of PCNA^{+} cells and DCX^{+} neuroblasts in the dorsal LSW of Fluoxetine-treated *Hes5::GFP*, *BLBP::mCherry* mice relative to vehicle-treated mice, 2 days after treatment. Bars represent mean ± SD of N=3 mice for vehicle and N=4 mice for Fluoxetine treatment (Relative PCNA^{+} cells: t=8.555 and df=5; Relative DCX^{+} cells: t=6.262 and df=5). Statistical significance was calculated by an unpaired t-test: **

P<0.01, ***P<0.001. (**G**) Bar plot showing the ratio of Hes5^{GFP+} and BLBP$^{mCherry+}$ cells in the dorsal LSW of Fluoxetine-treated *Hes5::GFP*, *BLBP::mCherry* mice relative to vehicle-treated mice, 19 days post-treatment. Bars represent mean ± SD of N=6 mice for both vehicle and Fluoxetine treatment (Relative Hes5^{GFP+} cells: t=3.138 and df=10; Relative BLBP$^{mCherry+}$ cells: t=3.681 and df=10). Statistical significance was calculated by an unpaired t-test: *P<0.05, ** P<0.01. cc, corpus callosum; sept, septum; dLS, dorsal lateral septum.

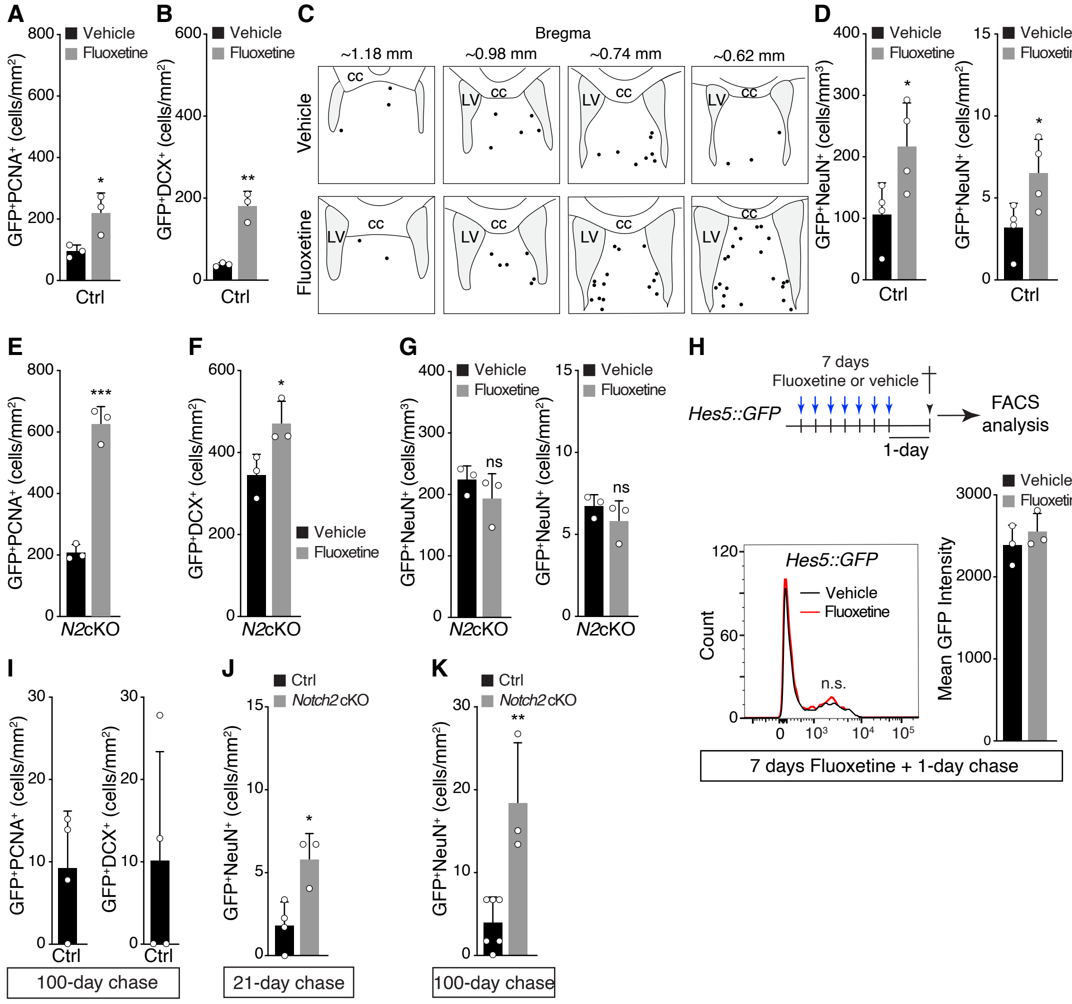

Fig. S9. Fluoxetine induces neurogenesis in the dorsal LSW. (**A**-**B**) Quantification of *Hes5::CreER^{T2}*-derived (GFP$^+$) mitotic cells (PCNA$^+$; A) and neuroblasts (DCX$^+$; B) in the dorsal LSW of Ctrl animals treated with Fluoxetine or vehicle. Bar plots depict cell densities per sectional area corresponding to the volumetric densities presented in Fig. 4D and E. PCNA$^+$ and DCX$^+$ cells were quantified in the dorsal LSW of both hemispheres on 3-4 coronal sections/mouse brain. Bars represent mean ± SD of N=3 mice per group. Statistical significance was calculated by an unpaired t-test: *P<0.05; **P<0.01. (**C**) Scheme of adult mouse brain coronal sections (Bregma 1.18 - 0.62 mm) in Ctrl mice after vehicle or Fluoxetine treatment. Black dots annotate the position of GFP$^+$NeuN$^+$ mature neurons. (**D**) Quantification of Hes5::CreER^{T2}-derived (GFP$^+$) mature neurons (NeuN$^+$) in the dorsal LSW of vehicle- or Fluoxetine-treated Ctrl animals, 14 days post-treatment. Bar plots are shown as volumetric densities (cells / mm^3) and as cell densities per sectional area (cells / mm^2). Bars represent mean ± SD of N=4 for Ctrl mice treated with vehicle or Fluoxetine. Statistical significance was calculated by an unpaired t-test: *P<0.05 (Ctrl, t=2.526 and df=6). (**E**-**F**) Quantification of *Hes5::CreER^{T2}*-derived (GFP$^+$) mitotic cells (PCNA$^+$; E) and neuroblasts (DCX$^+$; F) in the dorsal LSW of *Notch2* cKO animals treated with Fluoxetine or vehicle. Bar plots depict cell densities per sectional area corresponding to the volumetric densities presented in Fig. 4H and I. PCNA$^+$ and DCX$^+$ cells were quantified in the dorsal LSW of both hemispheres on 3-4 coronal sections/mouse brain. Bars represent mean ± SD of N=3 mice per group. Statistical significance was calculated by an unpaired t-test: *P<0.05; ***P<0.001. (**G**) Quantification of *Hes5::CreER^{T2}*-derived (GFP$^+$) mature neurons (NeuN$^+$) in the dorsal LSW of vehicle- or Fluoxetine-treated *Notch2* cKO animals, 14 days post-treatment. Bar plots are shown as volumetric densities (cells / mm^3) and as

cell densities per sectional area (cells / mm^2). Bars represent mean ± SD of N=3 mice for *Notch2* cKO mice treated with vehicle or Fluoxetine. Statistical significance was calculated by an unpaired t-test: ns, not significant (*Notch2* cKO, t=1.134 and df=4). (**H**) Notch signaling reporter animals (*Hes5::GFP*) were treated with Fluoxetine for 7 consecutive days. GFP intensity of dorsal LSW Hes5^{GFP+} cells was analyzed 1 day later by FACS. Quantification of dorsal LSW *Hes5::GFP* intensity by FACS. Bars represent mean ± SD of N=3 mice for both vehicle and Fluoxetine (t=0.8774 and df=4). Statistical significance was calculated by an unpaired t-test: ns, not significant. (**I**) Bar plots showing quantification of GFP$^+$PCNA$^+$ cells and GFP$^+$DCX$^+$ neuroblasts in the dorsal LSW of Ctrl animals, 100 days after Tamoxifen administration. Bars represent mean ± SD of N=4. Bar plots depict cell densities per sectional area corresponding to the volumetric densities presented in Fig. 5B. (**J**) Quantification of GFP$^+$NeuN$^+$ neurons in the LS of Ctrl and *Notch2* cKO animals, 21 days after Tamoxifen administration. Bar plot depicts cell densities per sectional area corresponding to the volumetric densities presented in Fig. 5C. Bars represent mean ± SD of N=4 Ctrl, and N=3 *Notch2* cKO. Statistical significance was calculated by an unpaired t-test: *P<0.05. (**K**) Quantification of GFP$^+$NeuN$^+$ neurons in the septum of Ctrl and *Notch2* cKO animals, 100 days after Tamoxifen administration. Bar plot depicts cell densities per sectional area corresponding to the volumetric densities presented in Fig. 5E. Bars represent mean ± SD of N=6 Ctrl, and N=3 *Notch2* cKO. Statistical significance was calculated by an unpaired t-test: **P<0.01. cc, corpus callosum; LV, lateral ventricle.

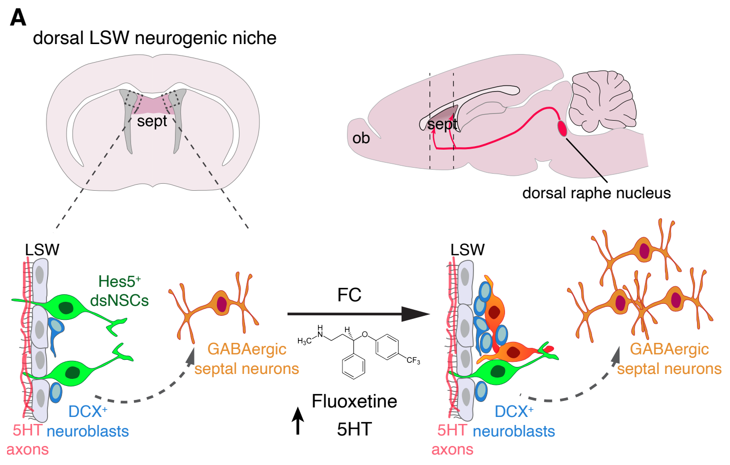

Fig. S10. Graphical summary. (**A**) Graphical summary of dorsal LSW neurogenic niche in close proximity with $5HT^{+}$ dorsal raphe afferent axons and the generation of local LS neurons. Dorsal LSW neurogenic potential is induced by FC and Fluoxetine treatment.

REAGENT or RESOURCE	SOURCE	IDENTIFIER
**Antibodies**		
Rabbit anti-β-Catenin	Sigma-Aldrich	Cat# C2206; RRID: AB_476831
Rat anti-BrdU	Bio-Rad	Cat# OBT0030; RRID: AB_609568
Mouse anti-Calbindin D28k	Swant	Cat# 300; RRID: AB_10000347
Rabbit anti-Calbindin D28k	Swant	Cat# CB38; RRID: AB_10000340
Rabbit anti-Calretinin	Swant	Cat# 7699/4; RRID: AB_2313763
Rat anti-CD31	BD Biosciences	Cat# 550274; RRID: AB_393571
Goat anti-Doublecortin	Santa Cruz	Cat# sc-8066; RRID: AB_2088494
Rabbit anti-dsRed	CloneTech Takara	Cat# 632496; RRID: AB_10013483
Mouse anti-GAD67	Millipore	Cat# MAB5406; RRID: AB_2278725
Mouse anti-GFAP	Sigma-Aldrich	Cat# G3893; RRID: AB_477010
Rabbit anti-GFAP	Sigma-Aldrich	Cat# G9269; RRID: AB_477035
Chicken anti-GFP	Aves labs	Cat# GFP-1020; RRID: AB_10000240
Rabbit anti-GFP	Invitrogen	Cat# A11122; RRID: AB_221569
Sheep anti-GFP	Bio-Rad	Cat# 4745-1051; RRID: AB_619712)
Mouse anti-NeuN	Millipore	Cat# MAB377; RRID: AB_2298772
Rat anti-NOTCH2	kind gift from H. Robson MacDonald ISREC, Lausanne	N/A
Mouse anti-Parvalbumin	Swant	Cat# 235; RRID: AB_10000343
Mouse anti-PCNA	Agilent	Cat# M0879; RRID: AB_2160651
Rabbit anti-Phospho-CREB (Ser133)	Cell Signaling Technology	Cat# 9198; RRID: AB_2561044
Rabbit anti-RBPj	Cell Signaling Technology	Cat# 5313; RRID: AB_2665555
Rabbit anti-Serotonin	Sigma-Aldrich	Cat# S5545; RRID: AB_477522
Rat anti-Somatostatin	Millipore	Cat# MAB354; RRID: AB_2255365)
Mouse anti-Tubulin Acetylated	Sigma-Aldrich	Cat# T6793; RRID: AB_477585
Donkey anti-chicken Alexa Fluor® 488	Jackson ImmunoResearch	Cat# 703-545-155; RRID: AB_2340375
Donkey anti-mouse Alexa Fluor® 488	Jackson ImmunoResearch	Cat# 715-546-151; RRID: AB_2340850
Donkey anti-rabbit Alexa Fluor® 488	Jackson ImmunoResearch	Cat# 711-545-152; RRID: AB_2313584
Donkey anti-sheep Alexa Fluor® 488	Jackson ImmunoResearch	Cat# 713-545-147; RRID: AB_2340745
Donkey anti-goat Cy3	Jackson ImmunoResearch	Cat# 705-165-147; RRID: AB_2307351
Donkey anti-mouse Cy3	Jackson ImmunoResearch	Cat# 715-165-151; RRID: AB_2315777
Donkey anti-rabbit Cy3	Jackson ImmunoResearch	Cat# 711-165-152; RRID: AB_2307443
Donkey anti-rat Cy3	Jackson ImmunoResearch	Cat# 712-166-153; RRID: AB_2340669
Donkey anti-goat Alexa Fluor® 647	Jackson ImmunoResearch	Cat# 705-605-147; RRID:AB_2340437)
Donkey anti-mouse Alexa Fluor® 647	Jackson ImmunoResearch	Cat# 715-605-150; RRID: AB_2340862

**Bacterial and virus strains**		
pAd/PLGFAPp-NLSCre-pA	Merkle et al (*71*)	N/A
pAAV-hSynapsin1-FLEx-axon-GCaMP6s	Broussard et al (*54*)	Addgene viral prep #112010-AAV5; http://n2t.net/addgene:112010; RRID:Addgene 112010
**Chemicals, peptides, and recombinant proteins**		
5-Bromo-2'-deoxyuridine (BrdU)	Sigma	Cat# B5002
BSA	Sigma	Cat# A3294
Corn oil	Sigma-Aldrich	Cat# C8267
L-Cysteine	Sigma-Aldrich	Cat# C7352
Cytosine arabinoside (AraC)	Sigma-Aldrich	Cat# C1768
DABCO	Sigma-Aldrich	Cat# D27802
DAPI	Roche	Cat# 10236276001
DNase I	Roche	Cat# 10104159001
Ethylen glycol	Merck	Cat# 1.00949.1000
Fluoxetine	Tocris	Cat# 0927
Glycerol	Sigma	Cat# G5516
GlycoBlue	Invitrogen	Cat# AM9515
L-15 Medium	Gibco	Cat# 31415-029
L-15 Medium no phenol red	Gibco	Cat# 21083-027
Normal Donkey Serum	Jackson ImmunoResearch	Cat# 017-000-121
Tissue-Tek® O.C.T. Compound	Sakura	Cat# 4583
Papain	Sigma	Cat# P3125-100MG
Paraformaldehyde	Carl Roth	Cat# 0335
Pen/Strep	Gibco	Cat# 15070063
Poly-L-lysine hydrobromide	Sigma	Cat# P9155
Sodium citrate	Sigma-Aldrich	Cat# W302600
Sodium phosphate dibasic dihydrate	Sigma-Aldrich	Cat# 30435
Sucrose	Sigma	Cat# 84100
Tamoxifen	Sigma	Cat# T5648
Triton X-100	PanReac AppliChem	Cat# A4975
TRIzol	Life Technologies	Cat# 15596026
Trypsin inhibitor Glycine max (Soybean)/Ovomucoid	Sigma	Cat# T6522
**Critical commercial assays**		
SuperScript III First-Strand Synthesis System for RT-PCR	Invitrogen	Cat# 18080051
**Deposited data**		
Neurogenic niche proteome	Kjell et al (*55*)	https://neuronicheproteome.org
**Experimental models: Organisms/strains**		
Mouse: *Hes5::GFP*	Basak and Taylor (*93*)	$Tg(Hes5\text{-}EGFP)^{2Vtlr}$
Mouse: *BLBP::mCherry*	Giachino et al (*56*)	$Tg(BLBP\text{-}mCherry)^{2Vtlr}$

Mouse: *Hes5::CreER*^{T2}	Lugert et al (*58*)	Tg(Hes5-cre/ERT2)^{2Vtlr}
Mouse: *Notch2::CreER*$^{T2\text{-}SAT}$	Fre et al (*94*)	Notch2$^{tm1.1(cre/ERT2)Sat}$
Mouse: *Rosa26R::GFP*	Tchorz et al (*95*)	Gt(ROSA)26Sor<tm1(CAG-EGFP)^{Vtlr}>
Mouse: *Rosa26R::tdTomato*	Madisen et al (*96*)	Gt(ROSA)26Sor$^{tm9(CAG\text{-}tdTomato)Hze}$; RRID:IMSR_JAX:007909
Mouse: *Notch1*$^{lox/lox}$	Radtke et al (*98*)	Notch1^{tm1Agt}
Mouse: *Notch2*$^{lox/lox}$	Besseyrias et al (*97*)	Notch2^{tm1Frad}
Mouse: *Rbpj*$^{lox/lox}$	Han et al (*99*)	Rbpj^{tm1Hon}
Mouse: *SERT::Cre*	Zhuang et al (*100*)	Slc6a4$^{tm1(cre)Xz}$; RRID:IMSR_JAX:014554
**Oligonucleotides**		
Primer: *5Htr1a*_forward GACAGGCGGCAACGATACT	This paper	N/A
Primer: *5Htr1a*_reverse CCAAGGAGCCGATGAGATAGTT	This paper	N/A
Primer: *5Htr1b*_forward CGCCGACGGCTACATTTAC	This paper	N/A
Primer: *5Htr1b*_reverse TAGCTTCCGGGTCCGATACA	This paper	N/A
Primer: *5Htr1d*_forward CACCCGCACCTGGAACTTT	This paper	N/A
Primer: *5Htr1d*_reverse AGTGGAGGGATGGAGATACAAA	This paper	N/A
Primer: *5Htr1f*_forward ATCAACTCCCTCGTGATCGC	This paper	N/A
Primer: *5Htr1f*_reverse ACACGTACAACAGATGATGTCG	This paper	N/A
Primer: *5Htr2a*_forward CGAAGCCTCGAACTGGACAAT	This paper	N/A
Primer: *5Htr2a*_reverse CCGCAATGGTGAGAATAATCACG	This paper	N/A
Primer: HTR2B_forward GAACAAAGCACAACTTCTGAGC	This paper	N/A
Primer: *5Htr2b*_reverse CCGCGAGTATCAGGAGAGC	This paper	N/A
Primer: *5Htr2c*_forward TGCTGGTGGGACTACTTGTCA	This paper	N/A
Primer: *5Htr2c*_reverse GACGCAGTTGAAAATAGCACATC	This paper	N/A
Primer: *5Htr4*_forward TGCCAGCCTTTGGTTTATAGG	This paper	N/A
Primer: *5Htr4*_reverse TTCCAGCCTTGCATTATGGGG	This paper	N/A
Primer: *5Htr5a*_forward ACCATCCTCAAGGTACGCAC	This paper	N/A
Primer: *5Htr5a*_reverse CAGAGCACGTCACATGCGAT	This paper	N/A
Primer: *5Htr5b*_forward GGTGGTGCTCTTCGTCTACTG	This paper	N/A
Primer: *5Htr5b*_reverse TGGAAGGTTACTGTTGCTCGG	This paper	N/A
Primer: *5Htr6*_forward CCAGCCTGCCTTATGTCCTC	This paper	N/A

Primer: *5Htr6*_reverse CTGGGCACCTGCAAGGTTT	This paper	N/A
Primer: *5Htr7*_forward CCGACCTCTACGGCCATCT	This paper	N/A
Primer: *5Htr7*_reverse TCTCGACTCTGCCATAGTTGAT	This paper	N/A
Primer: *5Htr3a*_forward CTGTGGCGATCACCGGAAG	This paper	N/A
Primer: *5Htr3a*_reverse GGCTGACTGCGTAGAATAAAGG	This paper	N/A
Primer: *5Htr3b*_forward GCATCGGGTCCCATCAGATA	This paper	N/A
Primer: *5Htr3b*_reverse CAGCACGGCAAGGTAGATTC	This paper	N/A
Primer: *Actb*_forward CAACGGCTCCGGCATGTGC	This paper	N/A
Primer: *Actb*_reverse CTCTTGCTCTGGGCCTCG	This paper	N/A
**Software and algorithms**		
Fiji	Schindelin et al (*104*)	https://imagej.net/Fiji
FlowJo	Becton, Dickinson & Company	https://www.flowjo.com/
GraphPad Prism 9	GraphPad	https://www.graphpad.com/scientific-software/prism/
Illustrator	Adobe	https://www.adobe.com/Illustrator
Omero	OME	https://www.openmicroscopy.org/about/
EthoVision 14 (Noldus)	Noldus	https://www.noldus.com/ethovision-xt
DeepLabCut	Mathis et al (*102*)	https://github.com/DeepLabCut/DeepLabCut

Table S1. ***KEY RESOURCES TABLE***

REFERENCES

1. S. Matsubara, T. Matsuda, K. Nakashima, Regulation of adult mammalian neural stem cells and neurogenesis by cell extrinsic and intrinsic factors. *Cells* **10**, 1145 (2021).

2. K. Obernier, A. Alvarez-Buylla, Neural stem cells: Origin, heterogeneity and regulation in the adult mammalian brain. *Development* **146**, dev156059 (2019).

3. F. Doetsch, I. Caille, D. A. Lim, J. M. Garcia-Verdugo, A. Alvarez-Buylla, Subventricular zone astrocytes are neural stem cells in the adult mammalian brain. *Cell* **97**, 703–716 (1999).

4. F. Doetsch, The glial identity of neural stem cells. *Nat. Neurosci.* **6**, 1127–1134 (2003).

5. K. L. Spalding, O. Bergmann, K. Alkass, S. Bernard, M. Salehpour, H. B. Huttner, E. Bostrom, I. Westerlund, C. Vial, B. A. Buchholz, G. Possnert, D. C. Mash, H. Druid, J. Frisen, Dynamics of hippocampal neurogenesis in adult humans. *Cell* **153**, 1219–1227 (2013).

6. A. Ernst, K. Alkass, S. Bernard, M. Salehpour, S. Perl, J. Tisdale, G. Possnert, H. Druid, J. Frisen, Neurogenesis in the striatum of the adult human brain. *Cell* **156**, 1072–1083 (2014).

7. E. C. Cope, E. Gould, Adult neurogenesis, glia, and the extracellular matrix. *Cell Stem Cell* **24**, 690–705 (2019).

8. M. Boldrini, C. A. Fulmore, A. N. Tartt, L. R. Simeon, I. Pavlova, V. Poposka, G. B. Rosoklija, A. Stankov, V. Arango, A. J. Dwork, R. Hen, J. J. Mann, Human hippocampal neurogenesis persists throughout aging. *Cell Stem Cell* **22**, 589–599.e5 (2018).

9. E. P. Moreno-Jimenez, M. Flor-Garcia, J. Terreros-Roncal, A. Rabano, F. Cafini, N. Pallas-Bazarra, J. Avila, M. Llorens-Martin, Adult hippocampal neurogenesis is abundant in neurologically healthy subjects and drops sharply in patients with Alzheimer's disease. *Nat. Med.* **25**, 554–560 (2019).

10. J. T. Goncalves, S. T. Schafer, F. H. Gage, Adult neurogenesis in the hippocampus: From stem cells to behavior. *Cell* **167**, 897–914 (2016).

11. I. Dumitru, M. Paterlini, M. Zamboni, C. Ziegenhain, S. Giatrellis, R. Saghaleyni, A. Bjorklund, K. Alkass, M. Tata, H. Druid, R. Sandberg, J. Frisen, Identification of proliferating neural progenitors in the adult human hippocampus. *Science* **389**, 58–63 (2025).

12. C. K. Tong, J. Chen, A. Cebrian-Silla, Z. Mirzadeh, K. Obernier, C. D. Guinto, L. H. Tecott, J. M. Garcia-Verdugo, A. Kriegstein, A. Alvarez-Buylla, Axonal control of the adult neural stem cell niche. *Cell Stem Cell* **14**, 500–511 (2014).

13. D. A. Berg, L. Belnoue, H. Song, A. Simon, Neurotransmitter-mediated control of neurogenesis in the adult vertebrate brain. *Development* **140**, 2548–2561 (2013).

14. B. A. Briones, E. Gould, "Adult neurogenesis and stress," in *Stress: Physiology, Biochemistry, and Pathology: Handbook of Stress Series*, G. Fink, Ed. (Academic Press, 2019), chap. 7, vol. 3, pp. 79–92.

15. M. Egeland, P. A. Zunszain, C. M. Pariante, Molecular mechanisms in the regulation of adult neurogenesis during stress. *Nat. Rev. Neurosci.* **16**, 189–200 (2015).

16. Y. S. Mineur, C. Belzung, W. E. Crusio, Functional implications of decreases in neurogenesis following chronic mild stress in mice. *Neuroscience* **150**, 251–259 (2007).

17. E. Gould, P. Tanapat, B. S. McEwen, G. Flugge, E. Fuchs, Proliferation of granule cell precursors in the dentate gyrus of adult monkeys is diminished by stress. *Proc. Natl. Acad. Sci. U.S.A.* **95**, 3168–3171 (1998).

18. D. J. David, B. A. Samuels, Q. Rainer, J. W. Wang, D. Marsteller, I. Mendez, M. Drew, D. A. Craig, B. P. Guiard, J. P. Guilloux, R. P. Artymyshyn, A. M. Gardier, C. Gerald, I. A. Antonijevic, E. D. Leonardo, R. Hen, Neurogenesis-dependent and -independent effects of fluoxetine in an animal model of anxiety/depression. *Neuron* **62**, 479–493 (2009).

19. C. Anacker, V. M. Luna, G. S. Stevens, A. Millette, R. Shores, J. C. Jimenez, B. Chen, R. Hen, Hippocampal neurogenesis confers stress resilience by inhibiting the ventral dentate gyrus. *Nature* **559**, 98–102 (2018).

20. J. S. Snyder, A. Soumier, M. Brewer, J. Pickel, H. A. Cameron, Adult hippocampal neurogenesis buffers stress responses and depressive behaviour. *Nature* **476**, 458–461 (2011).

21. L. Santarelli, M. Saxe, C. Gross, A. Surget, F. Battaglia, S. Dulawa, N. Weisstaub, J. Lee, R. Duman, O. Arancio, C. Belzung, R. Hen, Requirement of hippocampal neurogenesis for the behavioral effects of antidepressants. *Science* **301**, 805–809 (2003).

22. J. M. Encinas, A. Vaahtokari, G. Enikolopov, Fluoxetine targets early progenitor cells in the adult brain. *Proc. Natl. Acad. Sci. U.S.A.* **103**, 8233–8238 (2006).

23. B. A. Samuels, C. Anacker, A. Hu, M. R. Levinstein, A. Pickenhagen, T. Tsetsenis, N. Madronal, Z. R. Donaldson, L. J. Drew, A. Dranovsky, C. T. Gross, K. F. Tanaka, R. Hen, 5-HT1A receptors on mature dentate gyrus granule cells are critical for the antidepressant response. *Nat. Neurosci.* **18**, 1606–1616 (2015).

24. C. J. Harmer, R. S. Duman, P. J. Cowen, How do antidepressants work? New perspectives for refining future treatment approaches. *Lancet Psychiatry* **4**, 409–418 (2017).

25. C. N. Yohn, M. M. Gergues, B. A. Samuels, The role of 5-HT receptors in depression. *Mol. Brain* **10**, 28 (2017).

26. Y. F. Guzman, N. C. Tronson, V. Jovasevic, K. Sato, A. L. Guedea, H. Mizukami, K. Nishimori, J. Radulovic, Fear-enhancing effects of septal oxytocin receptors. *Nat. Neurosci.* **16**, 1185–1187 (2013).

27. F. Leroy, J. Park, A. Asok, D. H. Brann, T. Meira, L. M. Boyle, E. W. Buss, E. R. Kandel, S. A. Siegelbaum, A circuit from hippocampal CA2 to lateral septum disinhibits social aggression. *Nature* **564**, 213–218 (2018).

28. S. Shin, H. Pribiag, V. Lilascharoen, D. Knowland, X. Y. Wang, B. K. Lim, Drd3 signaling in the lateral septum mediates early life stress-induced social dysfunction. *Neuron* **97**, 195–208.e6 (2018).

29. X. Wu, W. Morishita, K. T. Beier, B. D. Heifets, R. C. Malenka, 5-HT modulation of a medial septal circuit tunes social memory stability. *Nature* **599**, 96–101 (2021).

30. R. Menon, T. Suss, V. E. M. Oliveira, I. D. Neumann, A. Bludau, Neurobiology of the lateral septum: Regulation of social behavior. *Trends Neurosci.* **45**, 27–40 (2022).

31. T. P. Sheehan, R. A. Chambers, D. S. Russell, Regulation of affect by the lateral septum: Implications for neuropsychiatry. *Brain Res. Brain Res. Rev.* **46**, 71–117 (2004).

32. Y. Takeuchi, A. J. Nagy, L. Barcsai, Q. Li, M. Ohsawa, K. Mizuseki, A. Berenyi, The medial septum as a potential target for treating brain disorders associated with oscillopathies. *Front. Neural Circuits* **15**, 701080 (2021).

33. A. Surget, C. Belzung, Adult hippocampal neurogenesis shapes adaptation and improves stress response: A mechanistic and integrative perspective. *Mol. Psychiatry* **27**, 403–421 (2022).

34. M. R. Drew, K. A. Huckleberry, Modulation of aversive memory by adult hippocampal neurogenesis. *Neurotherapeutics* **14**, 646–661 (2017).

35. A. Besnard, A. Sahay, Adult hippocampal neurogenesis, fear generalization, and stress. *Neuropsychopharmacology* **41**, 24–44 (2016).

36. C. Anacker, R. Hen, Adult hippocampal neurogenesis and cognitive flexibility—Linking memory and mood. *Nat. Rev. Neurosci.* **18**, 335–346 (2017).

37. K. A. Huckleberry, F. Shue, T. Copeland, R. A. Chitwood, W. Yin, M. R. Drew, Dorsal and ventral hippocampal adult-born neurons contribute to context fear memory. *Neuropsychopharmacology* **43**, 2487–2496 (2018).

38. R. J. Schloesser, H. K. Manji, K. Martinowich, Suppression of adult neurogenesis leads to an increased hypothalamo-pituitary-adrenal axis response. *Neuroreport* **20**, 553–557 (2009).

39. A. Sahay, K. N. Scobie, A. S. Hill, C. M. O'Carroll, M. A. Kheirbek, N. S. Burghardt, A. A. Fenton, A. Dranovsky, R. Hen, Increasing adult hippocampal neurogenesis is sufficient to improve pattern separation. *Nature* **472**, 466–470 (2011).

40. A. Surget, A. Tanti, E. D. Leonardo, A. Laugeray, Q. Rainer, C. Touma, R. Palme, G. Griebel, Y. Ibarguen-Vargas, R. Hen, C. Belzung, Antidepressants recruit new neurons to improve stress response regulation. *Mol. Psychiatry* **16**, 1177–1188 (2011).

41. D. O. Seo, M. A. Carillo, S. Chih-Hsiung Lim, K. F. Tanaka, M. R. Drew, Adult hippocampal neurogenesis modulates fear learning through associative and nonassociative mechanisms. *J. Neurosci.* **35**, 11330–11345 (2015).

42. C. A. Denny, N. S. Burghardt, D. M. Schachter, R. Hen, M. R. Drew, 4- to 6-week-old adult-born hippocampal neurons influence novelty-evoked exploration and contextual fear conditioning. *Hippocampus* **22**, 1188–1201 (2012).

43. J. M. Revest, D. Dupret, M. Koehl, C. Funk-Reiter, N. Grosjean, P. V. Piazza, D. N. Abrous, Adult hippocampal neurogenesis is involved in anxiety-related behaviors. *Mol. Psychiatry* **14**, 959–967 (2009).

44. N. S. Burghardt, D. E. Bush, B. S. McEwen, J. E. LeDoux, Acute selective serotonin reuptake inhibitors increase conditioned fear expression: Blockade with a 5-HT(2C) receptor antagonist. *Biol. Psychiatry* **62**, 1111–1118 (2007).

45. J. E. LeDoux, Emotion circuits in the brain. *Annu. Rev. Neurosci.* **23**, 155–184 (2000).

46. S. Maren, Neurobiology of Pavlovian fear conditioning. *Annu. Rev. Neurosci.* **24**, 897–931 (2001).

47. M. W. Hale, A. Shekhar, C. A. Lowry, Stress-related serotonergic systems: Implications for symptomatology of anxiety and affective disorders. *Cell. Mol. Neurobiol.* **32**, 695–708 (2012).

48. C. A. Marcinkiewcz, C. M. Mazzone, G. D'Agostino, L. R. Halladay, J. A. Hardaway, J. F. DiBerto, M. Navarro, N. Burnham, C. Cristiano, C. E. Dorrier, G. J. Tipton, C. Ramakrishnan, T.

Kozicz, K. Deisseroth, T. E. Thiele, Z. A. McElligott, A. Holmes, L. K. Heisler, T. L. Kash, Serotonin engages an anxiety and fear-promoting circuit in the extended amygdala. *Nature* **537**, 97–101 (2016).

49. B. M. Spannuth, M. W. Hale, A. K. Evans, J. L. Lukkes, S. Campeau, C. A. Lowry, Investigation of a central nucleus of the amygdala/dorsal raphe nucleus serotonergic circuit implicated in fear-potentiated startle. *Neuroscience* **179**, 104–119 (2011).

50. J. R. Awasthi, K. Tamada, E. T. N. Overton, T. Takumi, Comprehensive topographical map of the serotonergic fibers in the male mouse brain. *J. Comp. Neurol.* **529**, 1391–1429 (2021).

51. A. Dinopoulos, I. Dori, J. G. Parnavelas, Serotonergic innervation of the mature and developing lateral septum of the rat: A light and electron microscopic immunocytochemical analysis. *Neuroscience* **55**, 209–222 (1993).

52. K. Ishimura, Y. Takeuchi, K. Fujiwara, M. Tominaga, H. Yoshioka, T. Sawada, Quantitative analysis of the distribution of serotonin-immunoreactive cell bodies in the mouse brain. *Neurosci. Lett.* **91**, 265–270 (1988).

53. H. W. M. Steinbusch, M. A. Dolatkhah, D. A. Hopkins, Anatomical and neurochemical organization of the serotonergic system in the mammalian brain and in particular the involvement of the dorsal raphe nucleus in relation to neurological diseases. *Prog. Brain Res.* **261**, 41–81 (2021).

54. G. J. Broussard, Y. Liang, M. Fridman, E. K. Unger, G. Meng, X. Xiao, N. Ji, L. Petreanu, L. Tian, In vivo measurement of afferent activity with axon-specific calcium imaging. *Nat. Neurosci.* **21**, 1272–1280 (2018).

55. J. Kjell, J. Fischer-Sternjak, A. J. Thompson, C. Friess, M. J. Sticco, F. Salinas, J. Cox, D. C. Martinelli, J. Ninkovic, K. Franze, H. B. Schiller, M. Gotz, Defining the adult neural stem cell niche proteome identifies key regulators of adult neurogenesis. *Cell Stem Cell* **26**, 277–293.e8 (2020).

56. C. Giachino, O. Basak, S. Lugert, P. Knuckles, K. Obernier, R. Fiorelli, S. Frank, O. Raineteau, A. Alvarez-Buylla, V. Taylor, Molecular diversity subdivides the adult forebrain neural stem cell population. *Stem Cells* **32**, 70–84 (2014).

57. S. Lugert, O. Basak, P. Knuckles, U. Haussler, K. Fabel, M. Gotz, C. A. Haas, G. Kempermann, V. Taylor, C. Giachino, Quiescent and active hippocampal neural stem cells with distinct morphologies respond selectively to physiological and pathological stimuli and aging. *Cell Stem Cell* **6**, 445–456 (2010).

58. S. Lugert, M. Vogt, J. S. Tchorz, M. Muller, C. Giachino, V. Taylor, Homeostatic neurogenesis in the adult hippocampus does not involve amplification of Ascl1(high) intermediate progenitors. *Nat. Commun.* **3**, 670 (2012).

59. A. Lampada, V. Taylor, Notch signaling as a master regulator of adult neurogenesis. *Front. Neurosci.* **17**, 1179011 (2023).

60. O. Basak, C. Giachino, E. Fiorini, H. R. Macdonald, V. Taylor, Neurogenic subventricular zone stem/progenitor cells are Notch1-dependent in their active but not quiescent state. *J. Neurosci.* **32**, 5654–5666 (2012).

61. A. Engler, C. Rolando, C. Giachino, I. Saotome, A. Erni, C. Brien, R. Zhang, U. Zimber-Strobl, F. Radtke, S. Artavanis-Tsakonas, A. Louvi, V. Taylor, Notch2 signaling maintains NSC quiescence in the murine ventricular-subventricular zone. *Cell Rep.* **22**, 992–1002 (2018).

62. I. Imayoshi, M. Sakamoto, M. Yamaguchi, K. Mori, R. Kageyama, Essential roles of Notch signaling in maintenance of neural stem cells in developing and adult brains. *J. Neurosci.* **30**, 3489–3498 (2010).

63. H. Kawai, D. Kawaguchi, B. D. Kuebrich, T. Kitamoto, M. Yamaguchi, Y. Gotoh, S. Furutachi, Area-specific regulation of quiescent neural stem cells by Notch3 in the adult mouse subependymal zone. *J. Neurosci.* **37**, 11867–11880 (2017).

64. Y. Nyfeler, R. D. Kirch, N. Mantei, D. P. Leone, F. Radtke, U. Suter, V. Taylor, Jagged1 signals in the postnatal subventricular zone are required for neural stem cell self-renewal. *EMBO J.* **24**, 3504–3515 (2005).

65. R. Zhang, A. Engler, V. Taylor, Notch: An interactive player in neurogenesis and disease. *Cell Tissue Res.* **371**, 73–89 (2018).

66. J. Hatakeyama, Y. Bessho, K. Katoh, S. Ookawara, M. Fujioka, F. Guillemot, R. Kageyama, Hes genes regulate size, shape and histogenesis of the nervous system by control of the timing of neural stem cell differentiation. *Development* **131**, 5539–5550 (2004).

67. F. Doetsch, J. M. Garcia-Verdugo, A. Alvarez-Buylla, Regeneration of a germinal layer in the adult mammalian brain. *Proc. Natl. Acad. Sci. U.S.A.* **96**, 11619–11624 (1999).

68. J. M. Encinas, T. V. Michurina, N. Peunova, J. H. Park, J. Tordo, D. A. Peterson, G. Fishell, A. Koulakov, G. Enikolopov, Division-coupled astrocytic differentiation and age-related depletion of neural stem cells in the adult hippocampus. *Cell Stem Cell* **8**, 566–579 (2011).

69. R. Zhang, M. Boareto, A. Engler, A. Louvi, C. Giachino, D. Iber, V. Taylor, Id4 downstream of Notch2 maintains neural stem cell quiescence in the adult hippocampus. *Cell Rep.* **28**, 1485–1498.e6 (2019).

70. Z. Mirzadeh, F. T. Merkle, M. Soriano-Navarro, J. M. Garcia-Verdugo, A. Alvarez-Buylla, Neural stem cells confer unique pinwheel architecture to the ventricular surface in neurogenic regions of the adult brain. *Cell Stem Cell* **3**, 265–278 (2008).

71. F. T. Merkle, Z. Mirzadeh, A. Alvarez-Buylla, Mosaic organization of neural stem cells in the adult brain. *Science* **317**, 381–384 (2007).

72. F. T. Merkle, L. C. Fuentealba, T. A. Sanders, L. Magno, N. Kessaris, A. Alvarez-Buylla, Adult neural stem cells in distinct microdomains generate previously unknown interneuron types. *Nat. Neurosci.* **17**, 207–214 (2014).

73. M. C. Tiveron, C. Beclin, S. Murgan, S. Wild, A. Angelova, J. Marc, N. Core, A. de Chevigny, E. Herrera, A. Bosio, V. Bertrand, H. Cremer, Zic-proteins are repressors of dopaminergic forebrain fate in mice and *C. elegans*. *J. Neurosci.* **37**, 10611–10623 (2017).

74. A. Hai, L. X. Cai, T. Lee, V. S. Lelyveld, A. Jasanoff, Molecular fMRI of serotonin transport. *Neuron* **92**, 754–765 (2016).

75. C. Zhao, B. Eisinger, S. C. Gammie, Characterization of GABAergic neurons in the mouse lateral septum: A double fluorescence in situ hybridization and immunohistochemical study using tyramide signal amplification. *PLOS ONE* **8**, e73750 (2013).

76. G. Y. Wu, K. Deisseroth, R. W. Tsien, Activity-dependent CREB phosphorylation: Convergence of a fast, sensitive calmodulin kinase pathway and a slow, less sensitive mitogen-activated protein kinase pathway. *Proc. Natl. Acad. Sci. U.S.A.* **98**, 2808–2813 (2001).

77. T. Jungenitz, T. Radic, P. Jedlicka, S. W. Schwarzacher, High-frequency stimulation induces gradual immediate early gene expression in maturing adult-generated hippocampal granule cells. *Cereb. Cortex* **24**, 1845–1857 (2014).

78. G. E. Hoffman, W. W. Le, R. Abbud, W. S. Lee, M. S. Smith, Use of Fos-related antigens (FRAs) as markers of neuronal activity: FRA changes in dopamine neurons during proestrus, pregnancy and lactation. *Brain Res.* **654**, 207–215 (1994).

79. G. M. Singewald, A. Rjabokon, N. Singewald, K. Ebner, The modulatory role of the lateral septum on neuroendocrine and behavioral stress responses. *Neuropsychopharmacology* **36**, 793–804 (2011).

80. T. E. Anthony, N. Dee, A. Bernard, W. Lerchner, N. Heintz, D. J. Anderson, Control of stress-induced persistent anxiety by an extra-amygdala septohypothalamic circuit. *Cell* **156**, 522–536 (2014).

81. E. P. Azevedo, B. Tan, L. E. Pomeranz, V. Ivan, R. Fetcho, M. Schneeberger, K. R. Doerig, C. Liston, J. M. Friedman, S. A. Stern, A limbic circuit selectively links active escape to food suppression. *eLife* **9**, e58894 (2020).

82. K. Deng, L. Yang, J. Xie, H. Tang, G. S. Wu, H. R. Luo, Whole-brain mapping of projection from mouse lateral septal nucleus. *Biol. Open* **8**, bio043554 (2019).

83. Z. Chaker, C. Segalada, J. A. Kretz, I. E. Acar, A. C. Delgado, V. Crotet, A. E. Moor, F. Doetsch, Pregnancy-responsive pools of adult neural stem cells for transient neurogenesis in mothers. *Science* **382**, 958–963 (2023).

84. A. C. Delgado, A. R. Maldonado-Soto, V. Silva-Vargas, D. Mizrak, T. von Kanel, K. R. Tan, A. Paul, A. Madar, H. Cuervo, J. Kitajewski, C. S. Lin, F. Doetsch, Release of stem cells from quiescence reveals gliogenic domains in the adult mouse brain. *Science* **372**, 1205–1209 (2021).

85. S. Brummelte, L. A. Galea, Chronic high corticosterone reduces neurogenesis in the dentate gyrus of adult male and female rats. *Neuroscience* **168**, 680–690 (2010).

86. M. L. Lehmann, R. A. Brachman, K. Martinowich, R. J. Schloesser, M. Herkenham, Glucocorticoids orchestrate divergent effects on mood through adult neurogenesis. *J. Neurosci.* **33**, 2961–2972 (2013).

87. C. Anacker, A. Cattaneo, A. Luoni, K. Musaelyan, P. A. Zunszain, E. Milanesi, J. Rybka, A. Berry, F. Cirulli, S. Thuret, J. Price, M. A. Riva, M. Gennarelli, C. M. Pariante, Glucocorticoid-related molecular signaling pathways regulating hippocampal neurogenesis. *Neuropsychopharmacology* **38**, 872–883 (2013).

88. E. D. Kirby, S. E. Muroy, W. G. Sun, D. Covarrubias, M. J. Leong, L. A. Barchas, D. Kaufer, Acute stress enhances adult rat hippocampal neurogenesis and activation of newborn neurons via secreted astrocytic FGF2. *eLife* **2**, e00362 (2013).

89. G. Dagyte, E. A. Van der Zee, F. Postema, P. G. Luiten, J. A. Den Boer, A. Trentani, P. Meerlo, Chronic but not acute foot-shock stress leads to temporary suppression of cell proliferation in rat hippocampus. *Neuroscience* **162**, 904–913 (2009).

90. Y. J. Luo, H. Bao, A. Crowther, Y. D. Li, Z. K. Chen, D. S. Tart, B. Asrican, L. Zhang, J. Song, Sex-specific expression of distinct serotonin receptors mediates stress vulnerability of adult hippocampal neural stem cells in mice. *Cell Rep.* **43**, 114140 (2024).

91. P. C. Casarotto, M. Girych, S. M. Fred, V. Kovaleva, R. Moliner, G. Enkavi, C. Biojone, C. Cannarozzo, M. P. Sahu, K. Kaurinkoski, C. A. Brunello, A. Steinzeig, F. Winkel, S. Patil, S. Vestring, T. Serchov, C. Diniz, L. Laukkanen, I. Cardon, H. Antila, T. Rog, T. P. Piepponen, C. R. Bramham, C. Normann, S. E. Lauri, M. Saarma, I. Vattulainen, E. Castren, Antidepressant drugs act by directly binding to TRKB neurotrophin receptors. *Cell* **184**, 1299–1313.e19 (2021).

92. T. Fuchs, S. J. Jefferson, A. Hooper, P. H. Yee, J. Maguire, B. Luscher, Disinhibition of somatostatin-positive GABAergic interneurons results in an anxiolytic and antidepressant-like brain state. *Mol. Psychiatry* **22**, 920–930 (2017).

93. O. Basak, V. Taylor, Identification of self-replicating multipotent progenitors in the embryonic nervous system by high Notch activity and Hes5 expression. *Eur. J. Neurosci.* **25**, 1006–1022 (2007).

94. S. Fre, E. Hannezo, S. Sale, M. Huyghe, D. Lafkas, H. Kissel, A. Louvi, J. Greve, D. Louvard, S. Artavanis-Tsakonas, Notch lineages and activity in intestinal stem cells determined by a new set of knock-in mice. *PLOS ONE* **6**, e25785 (2011).

95. J. S. Tchorz, T. Suply, I. Ksiazek, C. Giachino, D. Cloetta, C. P. Danzer, T. Doll, A. Isken, M. Lemaistre, V. Taylor, B. Bettler, B. Kinzel, M. Mueller, A modified RMCE-compatible Rosa26 locus for the expression of transgenes from exogenous promoters. *PLOS ONE* **7**, e30011 (2012).

96. L. Madisen, T. A. Zwingman, S. M. Sunkin, S. W. Oh, H. A. Zariwala, H. Gu, L. L. Ng, R. D. Palmiter, M. J. Hawrylycz, A. R. Jones, E. S. Lein, H. Zeng, A robust and high-throughput Cre reporting and characterization system for the whole mouse brain. *Nat. Neurosci.* **13**, 133–140 (2010).

97. V. Besseyrias, E. Fiorini, L. J. Strobl, U. Zimber-Strobl, A. Dumortier, U. Koch, M. L. Arcangeli, S. Ezine, H. R. Macdonald, F. Radtke, Hierarchy of Notch-Delta interactions promoting T cell lineage commitment and maturation. *J. Exp. Med.* **204**, 331–343 (2007).

98. F. Radtke, A. Wilson, G. Stark, M. Bauer, J. van Meerwijk, H. R. MacDonald, M. Aguet, Deficient T cell fate specification in mice with an induced inactivation of Notch1. *Immunity* **10**, 547–558 (1999).

99. H. Han, K. Tanigaki, N. Yamamoto, K. Kuroda, M. Yoshimoto, T. Nakahata, K. Ikuta, T. Honjo, Inducible gene knockout of transcription factor recombination signal binding protein-J reveals its essential role in T versus B lineage decision. *Int. Immunol.* **14**, 637–645 (2002).

100. X. Zhuang, J. Masson, J. A. Gingrich, S. Rayport, R. Hen, Targeted gene expression in dopamine and serotonin neurons of the mouse brain. *J. Neurosci. Methods* **143**, 27–32 (2005).

101. C. Giachino, V. Taylor, Lineage analysis of quiescent regenerative stem cells in the adult brain by genetic labelling reveals spatially restricted neurogenic niches in the olfactory bulb. *Eur. J. Neurosci.* **30**, 9–24 (2009).

102. A. Mathis, P. Mamidanna, K. M. Cury, T. Abe, V. N. Murthy, M. W. Mathis, M. Bethge, DeepLabCut: Markerless pose estimation of user-defined body parts with deep learning. *Nat. Neurosci.* **21**, 1281–1289 (2018).

103. K. B. J. Franklin, G. Paxinos, *The Mouse Brain in Stereotaxic Coordinates* (Academic Press, 1997).

104. J. Schindelin, I. Arganda-Carreras, E. Frise, V. Kaynig, M. Longair, T. Pietzsch, S. Preibisch, C. Rueden, S. Saalfeld, B. Schmid, J. Y. Tinevez, D. J. White, V. Hartenstein, K. Eliceiri, P. Tomancak, A. Cardona, Fiji: An open-source platform for biological-image analysis. *Nat. Methods* **9**, 676–682 (2012).
